# Supplementary figures and images for: Ubx-Collier signaling cascade maintains blood progenitors in the posterior lobes of the Drosophila larval lymph gland
Source: PLoS Genet. 2021 Aug 9;17(8):e1009709. doi: 10.1371/journal.pgen.1009709 (PMC8376192; doi:10.1371/journal.pgen.1009709)

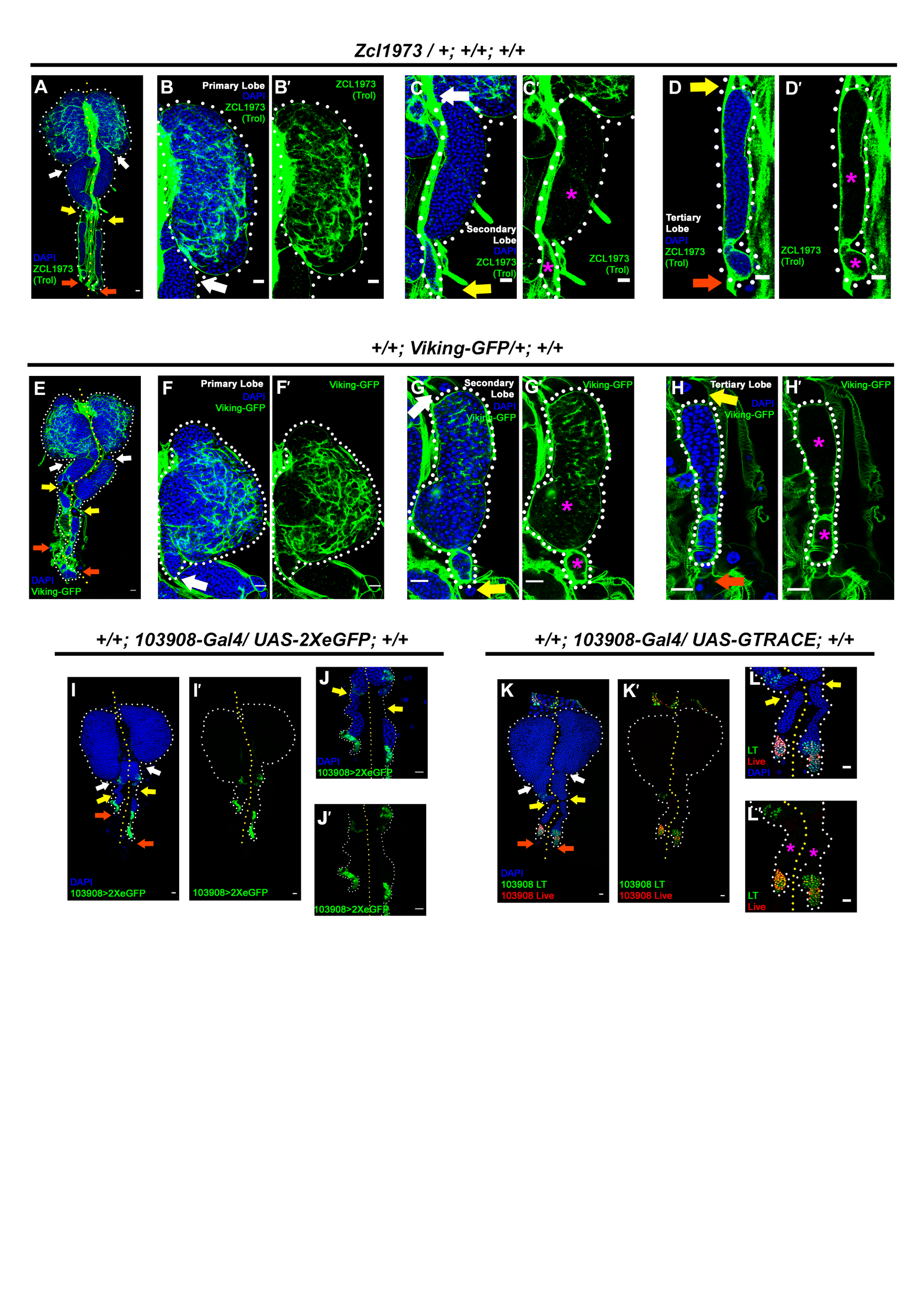

Supplement: S1 Fig — (A-D′.) Trol marks the progenitors and surrounds the niche in the primary lobe (B-B′). Within the posterior lobes, Trol expression creates a large anterior and a small posterior domain (C-D′, magenta asterisk). (E-H′.) Viking reporter expression marks the periphery of the primary lobe (F-F′) and analogous to Trol, divides the secondary and tertiary lobes into two domains (G-H′, magenta asterisk). (I-J′.) The 103908-Gal4 expression is restricted to the posterior domain of both the secondary and tertiary lobe. (K-L′.) 103908-Gal4 does not lineage trace to the anterior half of tertiary lobes (magenta asterisk). In all panels, lymph glands are dissected from third instar larvae (96hr after egg hatching, AEH). Arrows are pointed towards the intercalated pericardial cells within lymph gland lobes. The white arrow marks the pericardial cell between primary and secondary lobes; yellow between secondary and tertiary, and orange marks the last pericardial cell at the end of the LG organ after the tertiary lobe. White dotted lines mark the lymph gland boundary, while the yellow dotted line marks the aorta, which is between the bi-lobed lymph gland. The genotype of the larvae and labeling on the tissue is described in the panels. Scale bars: 20 μm. (TIF) [file pgen.1009709.s001.tif]

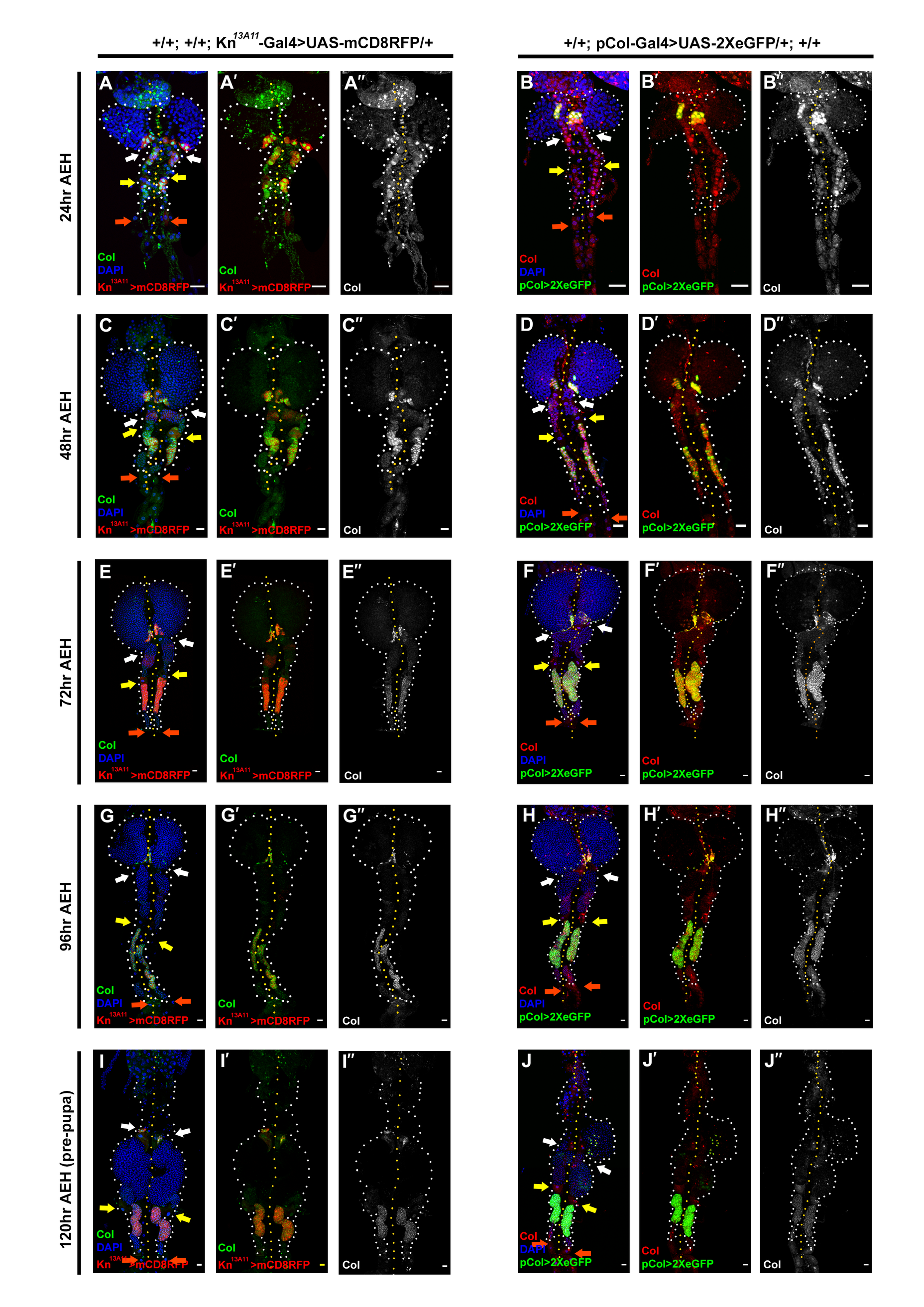

Supplement: S2 Fig — (A-B′′.) At 24hr AEH, the entire posterior lobes comprise a single stripe of Col expressing blood cells (marked by antibody) that co-localizes with Kn13A11-Gal4 but not pCol-Gal4. Col protein is high in primary lobe PSC but low in medullary zone. (C-F′′.) From 48–72 hr AEH, the high Col expression is restricted in the tertiary lobe co-localizing with both Kn13A11-Gal4 and pCol-Gal4, while a basal Col antibody labelling is observed in secondary lobes. (G-H′′.) At 96hr AEH, high Col expression prevails in the tertiary lobe co-localizing with Kn13A11-Gal4 and pCol-Gal4. However, faint Col antibody labelling is detectable in the posterior cells of secondary lobes. (I-J′′.) By 120 hr, even when the primary lobe dissociates, the tertiary lobes express high levels of Col that co-localizes with both the drivers. Larval staging is mentioned in the corresponding panels. Arrows are pointed towards the intercalated pericardial cells within lymph gland lobes. The white arrow marks the pericardial cell between primary and secondary lobes; yellow between secondary and tertiary, and orange marks the last pericardial cell at the end of the LG organ after the tertiary lobe. White dotted lines mark the lymph gland boundary, while the yellow dotted line marks the aorta, which is between the bi-lobed lymph gland. The genotype of the larvae and labeling on the tissue is described in the panels. Scale bars: 20 μm. (TIF) [file pgen.1009709.s002.tif]

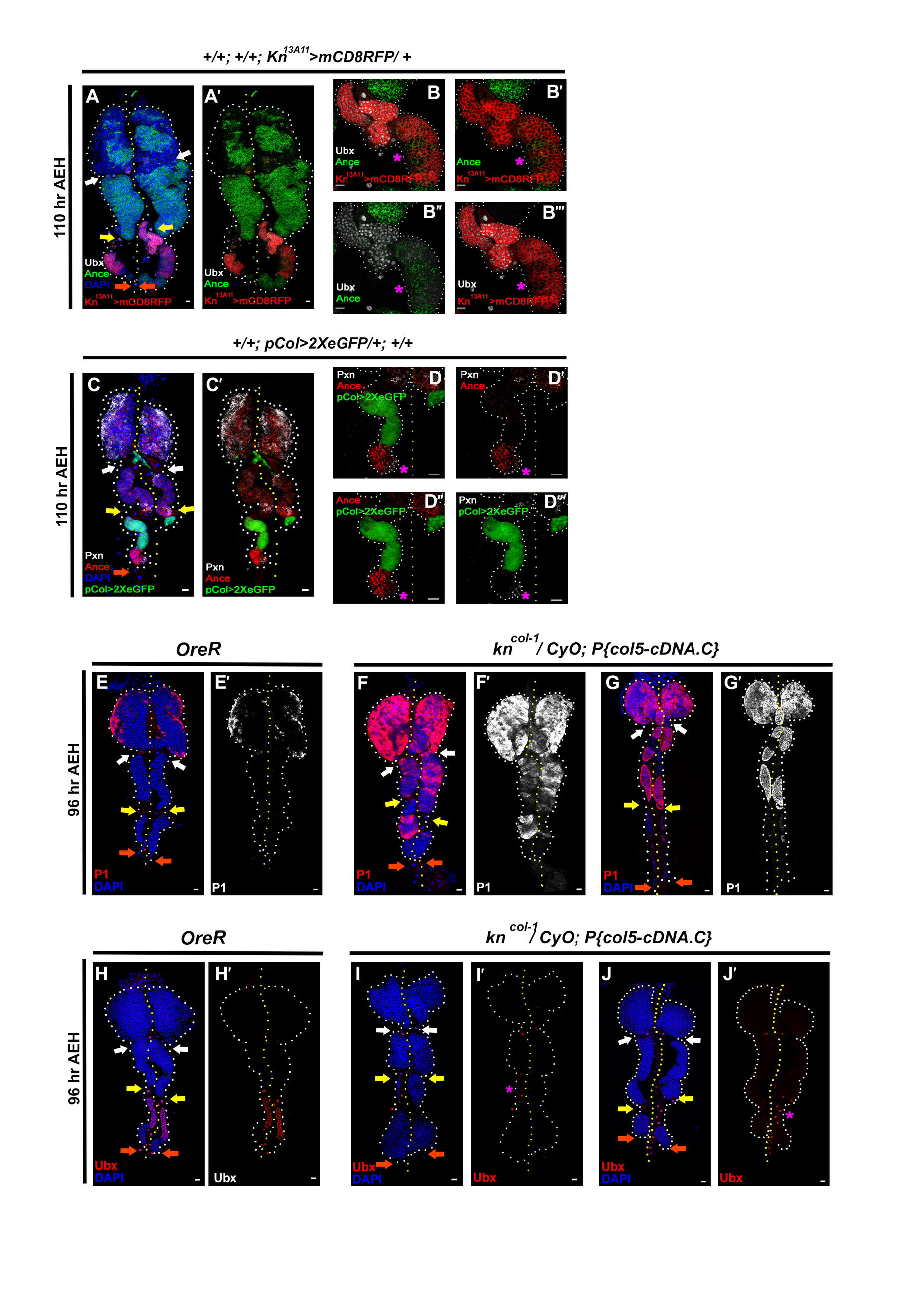

Supplement: S3 Fig — (A-B′′′.) At 110hr AEH, the Collier domain in the anterior half of the tertiary lobe harbours both Ubx expressing cells (high Col, Ubx positive) and progenitors (low Col, Ance positive: magenta asterisk). (C-D′′′.) At 110hr AEH, Pxn expression can be detected in the Collier negative domain of the tertiary lobe (magenta asterisk). (E-G′.) In Col loss, posterior lobes undergo ectopic differentiation (F-G′) in comparison to control (E-E′). (H-J′.) The loss of Ubx from posterior lobes is observed in Col mutants (I- J′) compared to controls (H-H′). Ubx expression, however, is still unperturbed in cardioblasts (magenta asterisk). In all panels, lymph glands are dissected from third instar larvae (96hr after egg hatching, AEH) unless otherwise mentioned. Arrows are pointed towards the intercalated pericardial cells within lymph gland lobes. The white arrow marks the pericardial cell between primary and secondary lobes; yellow between secondary and tertiary, and orange marks the last pericardial cell at the end of the LG organ after the tertiary lobe. White dotted lines mark the lymph gland boundary, while the yellow dotted line marks the aorta, which is between the bi-lobed lymph gland. The genotype of the larvae and labeling on the tissue is described in the panels. Scale bars: 20 μm. (TIF) [file pgen.1009709.s003.tif]

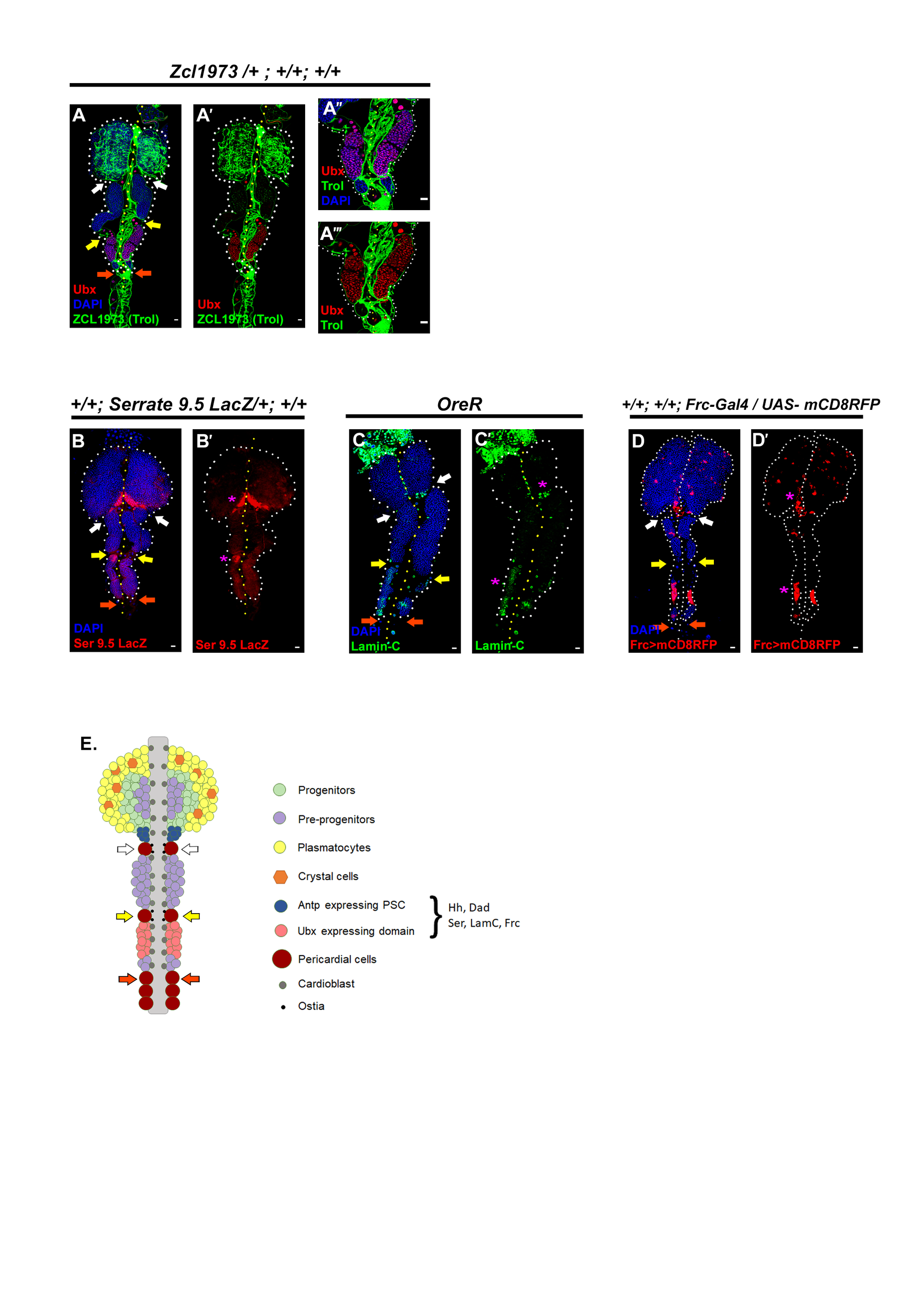

Supplement: S4 Fig — (A-A′′′) Trol compartmentalizes the Ubx domain. (B-D′) The anterior half of the tertiary lobe expresses Serrate visualized by Ser9.5LacZ (B-B′), LaminC (C-C′), and Fringe Connection (D- D′), all marked by a magenta asterisk. (E) Model summarizing the above findings and of Figs 3 and S4. In all panels, lymph glands are dissected from third instar larvae (96hr after egg hatching, AEH) unless otherwise mentioned. Arrows are pointed towards the intercalated pericardial cells within lymph gland lobes. The white arrow marks the pericardial cell between primary and secondary lobes; yellow between secondary and tertiary, and orange marks the last pericardial cell at the end of the LG organ after the tertiary lobe. White dotted lines mark the lymph gland boundary, while the yellow dotted line marks the aorta, which is between the bi-lobed lymph gland. The genotype of the larvae and labeling on the tissue is described in the panels. Scale bars: 20 μm. (TIF) [file pgen.1009709.s004.tif]

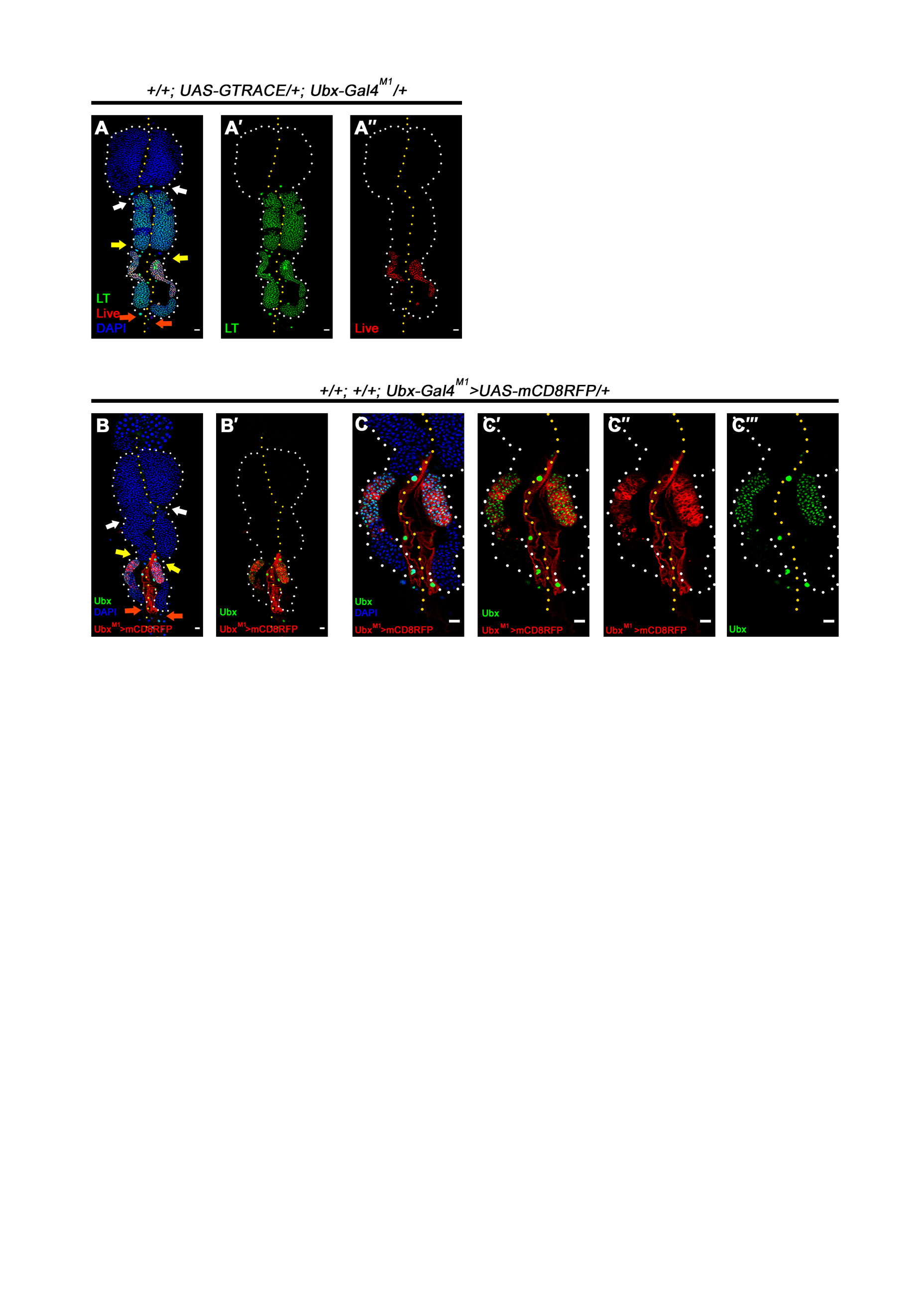

Supplement: S5 Fig — (A-A′′) Activation of Lineage tracing construct throughout development by Ubx-Gal4M1 marks the entire posterior lobes (A′), live expression is, however, restricted to the anterior half of tertiary lobes (A′′) at 96hr AEH. (B-C′′′) Ubx-Gal4M1 live expression in the tertiary lobe co-localizes with the Ubx antibody at 96hr AEH(B-B′) and zoomed in images (C-C′′′). In all panels, lymph glands are dissected from third instar larvae (96hr after egg hatching, AEH). Arrows are pointed towards the intercalated pericardial cells within lymph gland lobes. The white arrow marks the pericardial cell between primary and secondary lobes; yellow between secondary and tertiary, and orange marks the last pericardial cell at the end of the LG organ after the tertiary lobe. White dotted lines mark the lymph gland boundary, while the yellow dotted line marks the aorta, which is between the bi-lobed lymph gland. The genotype of the larvae and labeling on the tissue is described in the panels. Scale bars: 20 μm. (TIF) [file pgen.1009709.s005.tif]

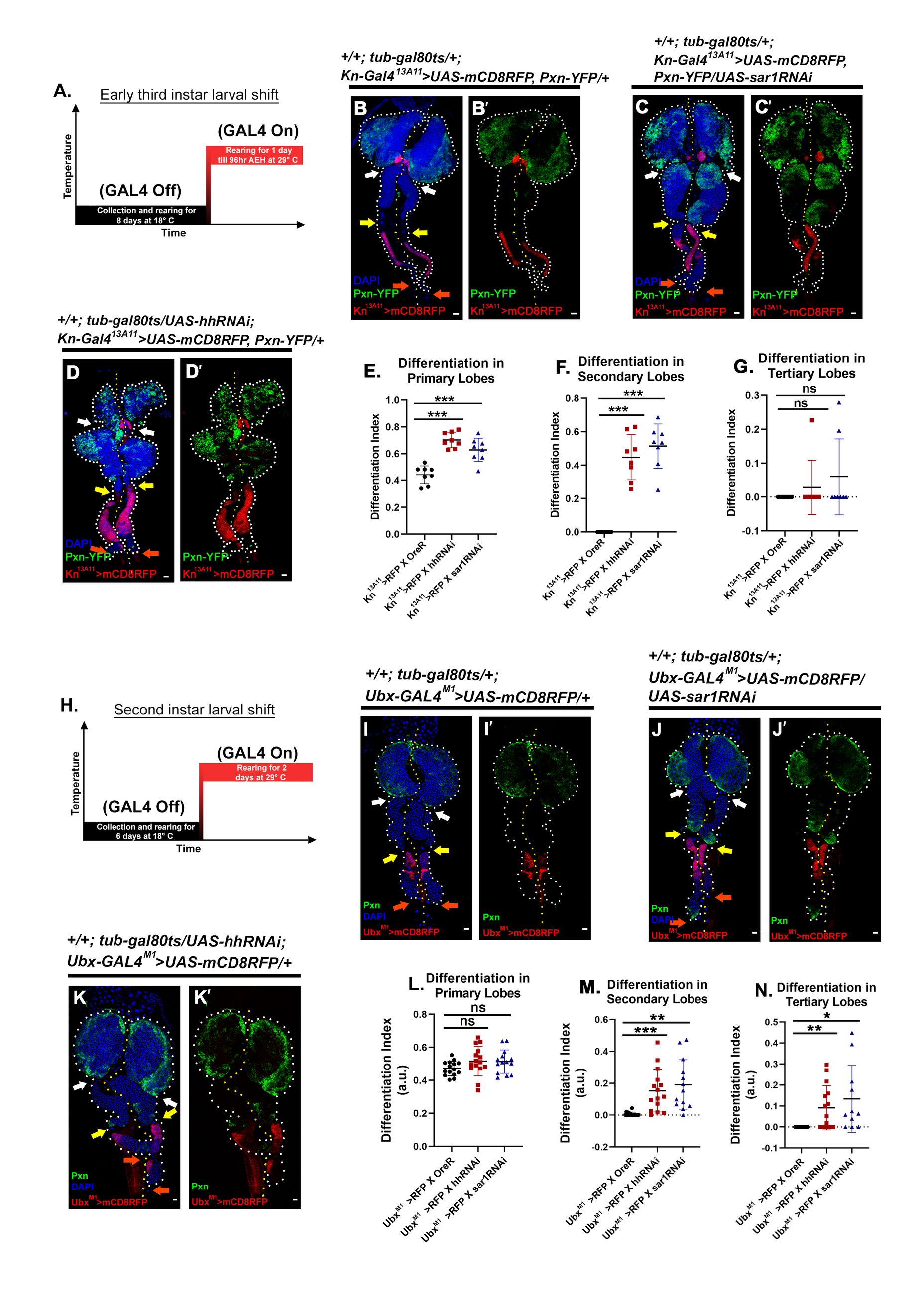

Supplement: S6 Fig — (A-D′.) Following the timeline in A, Kn13A11-Gal4 was employed to downregulate sar1 (C-C′) and hh (D-D′) from the Ubx zone which resulted in increased differentiation (Pxn) compared to control (B-B′).(E.) Quantification of B-D′, downregulation of sar1 (n = 8, P-value = 3.51E-04) and hh (n = 8, P-value = 1.10849E-06) for primary lobes.(F.) Quantification of B-D′, downregulation of sar1 (n = 8, P-value = 1.14317E-05) and hh (n = 8, P-value = 3.53517E-05) in the secondary lobes. (G.) Quantification of B-D′, downregulation of sar1 (n = 8, P-value = 0.1779) and hh (n = 8, P-value = 0.3506) in tertiary lobe progenitors. (H-K′.) Following the timeline in H, posterior lobe specific driver Ubx-Gal4M1 was employed to downregulate sar1 (J-J′) and hh (K-K′) from the Ubx zone resulted in increased differentiation (Pxn) compared to control (I-I′). (L.) Quantification of I-K′, downregulation of sar1 (Control: n = 15, sar1-RNAi: n = 14; P-value = 0.0719) and hh (Control: n = 15, hh-RNAi: n = 15; P-value = 0.0970) for primary lobes. (M.) Quantification of I-K′, downregulation of sar1 (Control: n = 15, sar1-RNAi: n = 13; P-value = 1.20E-03) and hh (Control: n = 15, hh-RNAi: n = 15; P-value = 7.70E-04) in the secondary lobes. (N.) Quantification of I-K′, downregulation of sar1 (Control: n = 15, sar1-RNAi: n = 11; P-value = 0.0190) and hh (Control: n = 15, hh-RNAi: n = 15; P-value = 4.66E-03) in tertiary lobe progenitors. In all panels, lymph glands are dissected from third instar larvae (96hr after egg hatching, AEH). Arrows are pointed towards the intercalated pericardial cells within lymph gland lobes. The white arrow marks the pericardial cell between primary and secondary lobes; yellow between secondary and tertiary, and orange marks the last pericardial cell at the end of the LG organ after the tertiary lobe. White dotted lines mark the lymph gland boundary, while the yellow dotted line marks the aorta, which is between the bi-lobed lymph gland. The genotype of the larvae and lab [file pgen.1009709.s006.tif]

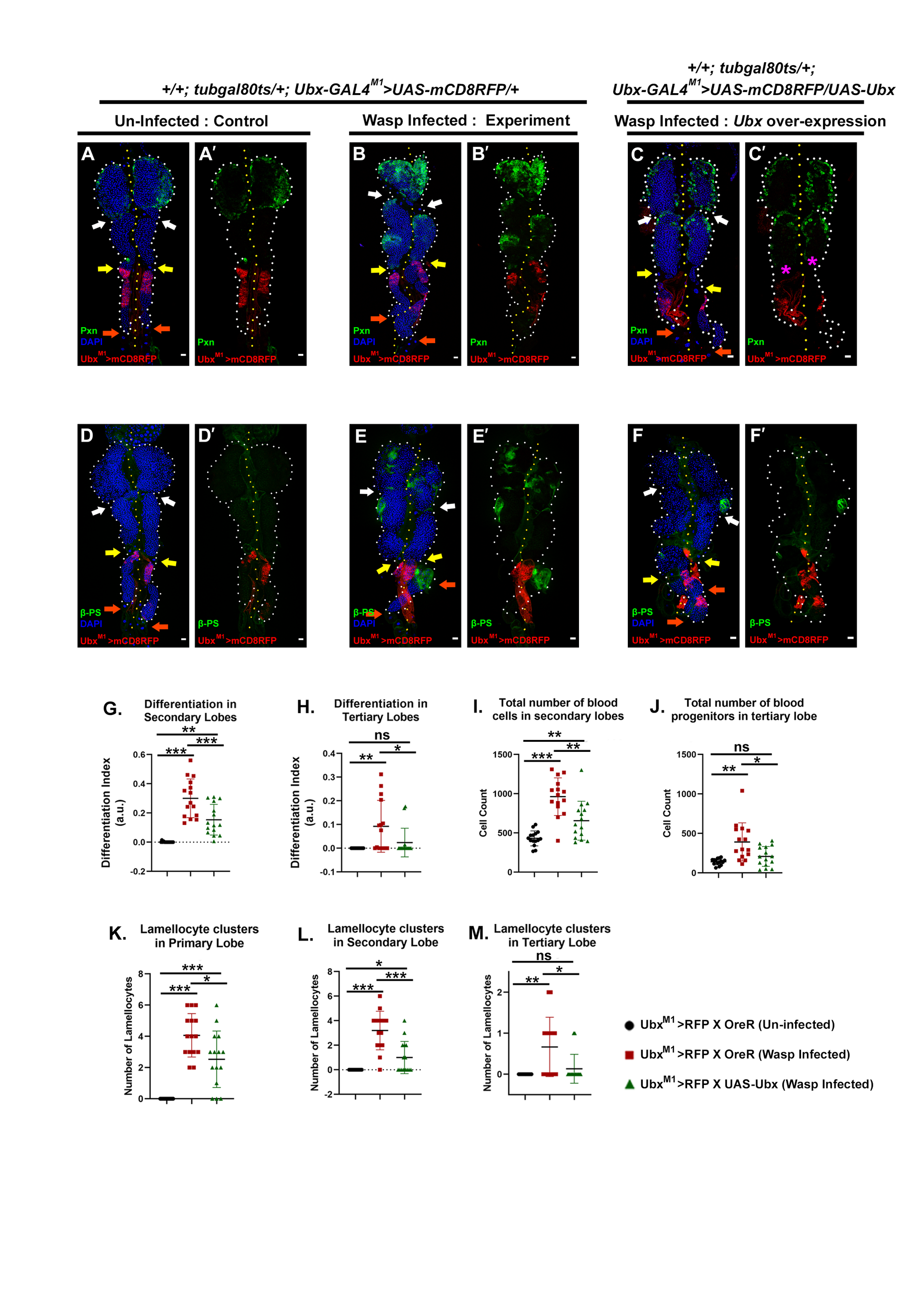

Supplement: S7 Fig — (A-C′.) Ectopic differentiation (Pxn) is observed upon wasp infection in primary and secondary lobes (B-B′) compared to uninfected control (A-A′). Overexpression of Ubx from the tertiary lobe utilizing posterior lobe specific driver Ubx-Gal4M1 suppresses the immune response from the posterior lobes (C-C′, magenta asterisk). (D-F′.) Lamellocyte induction (β-PS) is observed upon wasp infection in primary and secondary lobes (E-E′) compared to uninfected control (D-D′). Overexpression of Ubx from the tertiary lobe utilizing posterior lobe specific driver Ubx-Gal4M1 reduces the number of lamellocytes formed in the posterior lobes (F-F′). (G.) Quantification of the differentiation index of secondary lobe (A-C′.) {Uninfected vs Infected: n = 15, P-value = 4.676E-07}, {Uninfected vs Ubx overexpression: n = 15, P-value = 6.341E-05} and {Infected vs Ubx overexpression: n = 15, P-value = 2.342E-03}. (H.) Quantification of the differentiation index of tertiary lobe (A-C′.) {Uninfected vs Infected: n = 15, P-value = 5.558E-03}, {Uninfected vs Ubx overexpression: n = 15, P-value = 0.146} and {Infected vs Ubx overexpression: n = 15, P-value = 0.0455}. (I.) Quantification of total hemocyte pool in secondary lobe (A-F′) {Uninfected vs Infected: n = 15, P-value = 2.20E-07}, {Uninfected vs Ubx overexpression: n = 15, P-value = 4.19E-03} and {Infected vs Ubx overexpression: n = 15, P-value = 1.855E-03}. (J.) Quantification of the progenitor pool in tertiary lobe (A-F′.) {Uninfected vs Infected: n = 15, P-value = 1.283E-03}, {Uninfected vs Ubx overexpression: n = 15, P-value = 0.0652} and {Infected vs Ubx overexpression: n = 15, P-value = 0.0164}. (K.) Quantification of lamellocytes in primary lobe (D-F′) {Uninfected vs Infected: n = 15, P-value = 1.893E-08}, {Uninfected vs Ubx overexpression n = 15, P-value = 8.891E-05} and {Infected vs Ubx overexpression: n = 15, P-value = 0.0148}. (L.) Quantification of lamellocytes in secondary lobe (D-F′) {Uninfected vs Infected: n = 15, P-value = [file pgen.1009709.s007.tif]

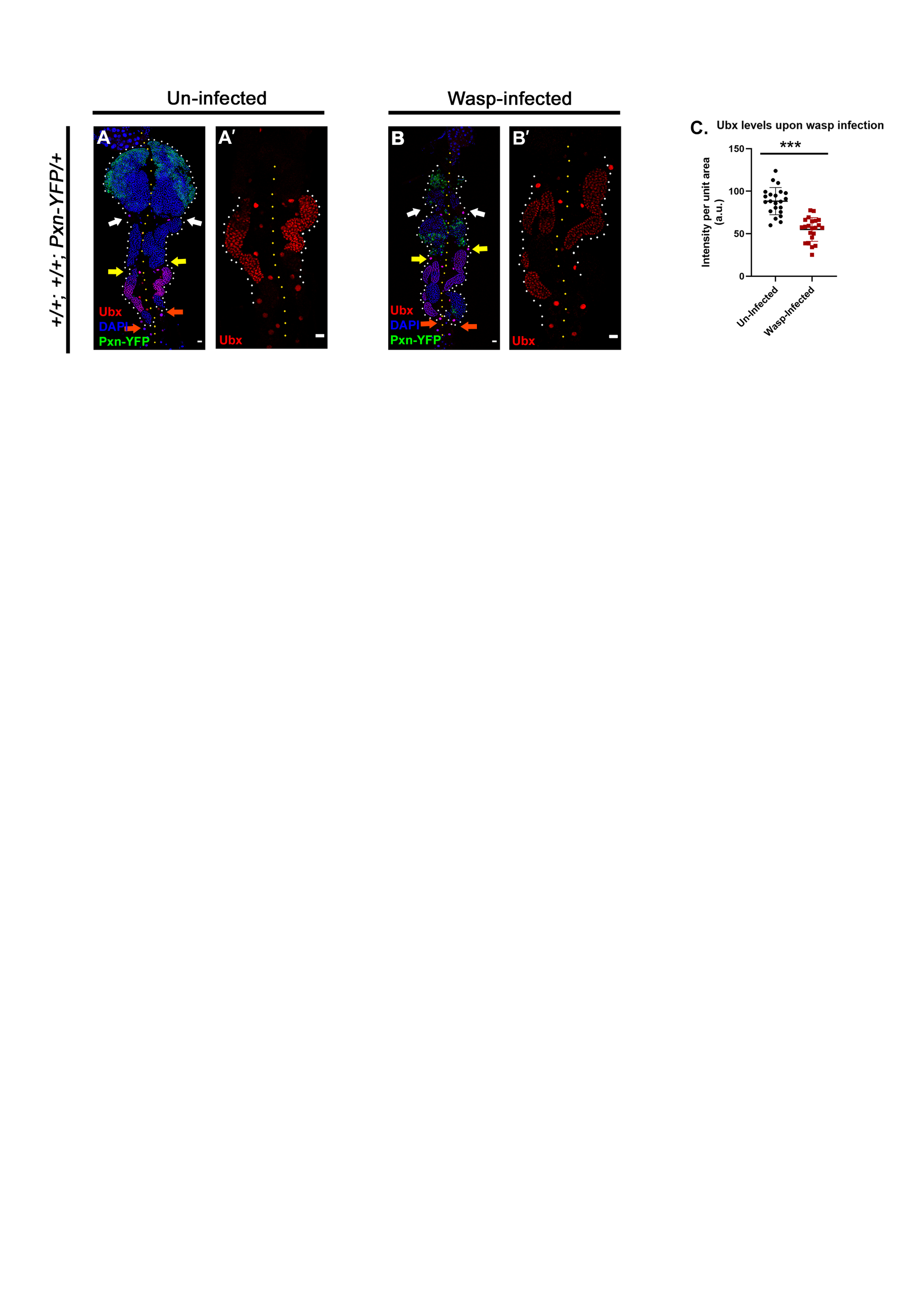

Supplement: S8 Fig — (A-B′.) Ubx levels drop substantially upon wasp infection induced immune challenge (B-B′) in comparison to un-infected control (A-A′). Note that there is no change in Ubx levels of neighbouring pericardial cells and cardioblasts. (C.) Statistical analysis reveals the significance of drop in Ubx levels (n = 22, P-value = 5.011E-09). In all panels, lymph glands are dissected from third instar larvae (96hr after egg hatching, AEH). Arrows are pointed towards the intercalated pericardial cells within lymph gland lobes. The white arrow marks the pericardial cell between primary and secondary lobes; yellow between secondary and tertiary, and orange marks the last pericardial cell at the end of the LG organ after the tertiary lobe. White dotted lines mark the lymph gland boundary, while the yellow dotted line marks the aorta, which is between the bi-lobed lymph gland. The genotype of the larvae and labeling on the tissue is described in the panels. Scale bars: 20 μm. Two-tailed unpaired Student’s t-test was performed for calculating statistical significance. Data are mean±s.d. *P<0.05, **P<0.001 and ***P<0.0001. (TIF) [file pgen.1009709.s008.tif]

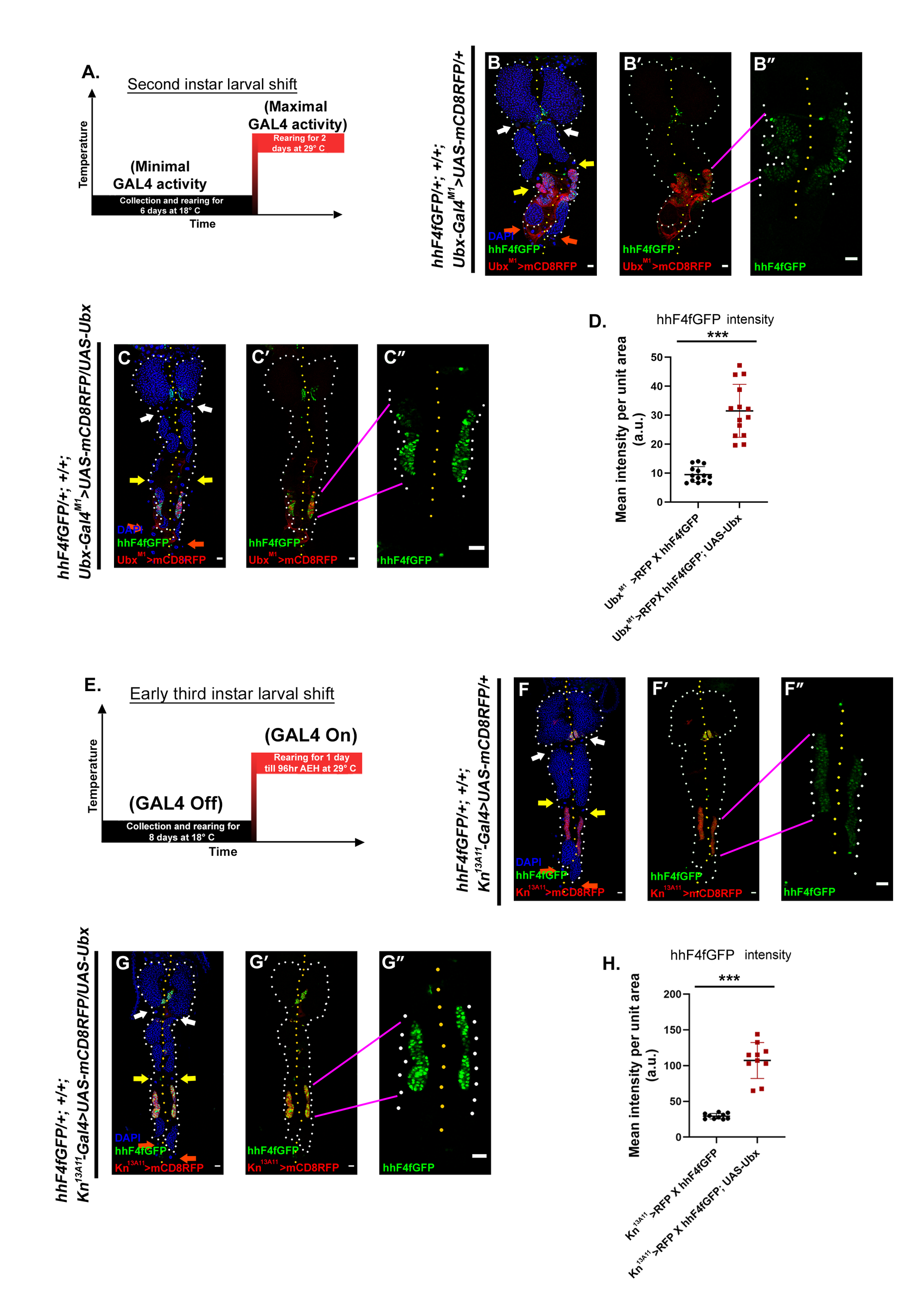

Supplement: S9 Fig — (A-C′′) Upregulation of Ubx via posterior lobe specific driver Ubx-Gal4M1 (C-C′′), following the scheme in A, leads to a substantial increase in hedgehog levels in comparison to control (B-B′′). (D.) Quantification of (B-C′′) in terms of hhF4fGFP signal (n = 14, P-value = 2.868E-07). (E-G′′) Upregulation of Ubx via Kn13A11-Gal4 (G-G′′), following the scheme in E, leads to a significant increase in hedgehog levels in comparison to control (F-F′′). (H.) Quantification of (F-G′′) in terms of hhF4fGFP signal (n = 10, P-value = 3.277E-06). In all panels, lymph glands are dissected from third instar larvae (96hr after egg hatching, AEH). Arrows are pointed towards the intercalated pericardial cells within lymph gland lobes. The white arrow marks the pericardial cell between primary and secondary lobes; yellow between secondary and tertiary, and orange marks the last pericardial cell at the end of the LG organ after the tertiary lobe. White dotted lines mark the lymph gland boundary, while the yellow dotted line marks the aorta, which is between the bi-lobed lymph gland,. The genotype of the larvae and labeling on the tissue is described in the panels. Scale bars: 20 μm. Two-tailed unpaired Student’s t-test was performed for calculating statistical significance. Data are mean±s.d. *P<0.05, **P<0.001 and ***P<0.0001. (TIF) [file pgen.1009709.s009.tif]

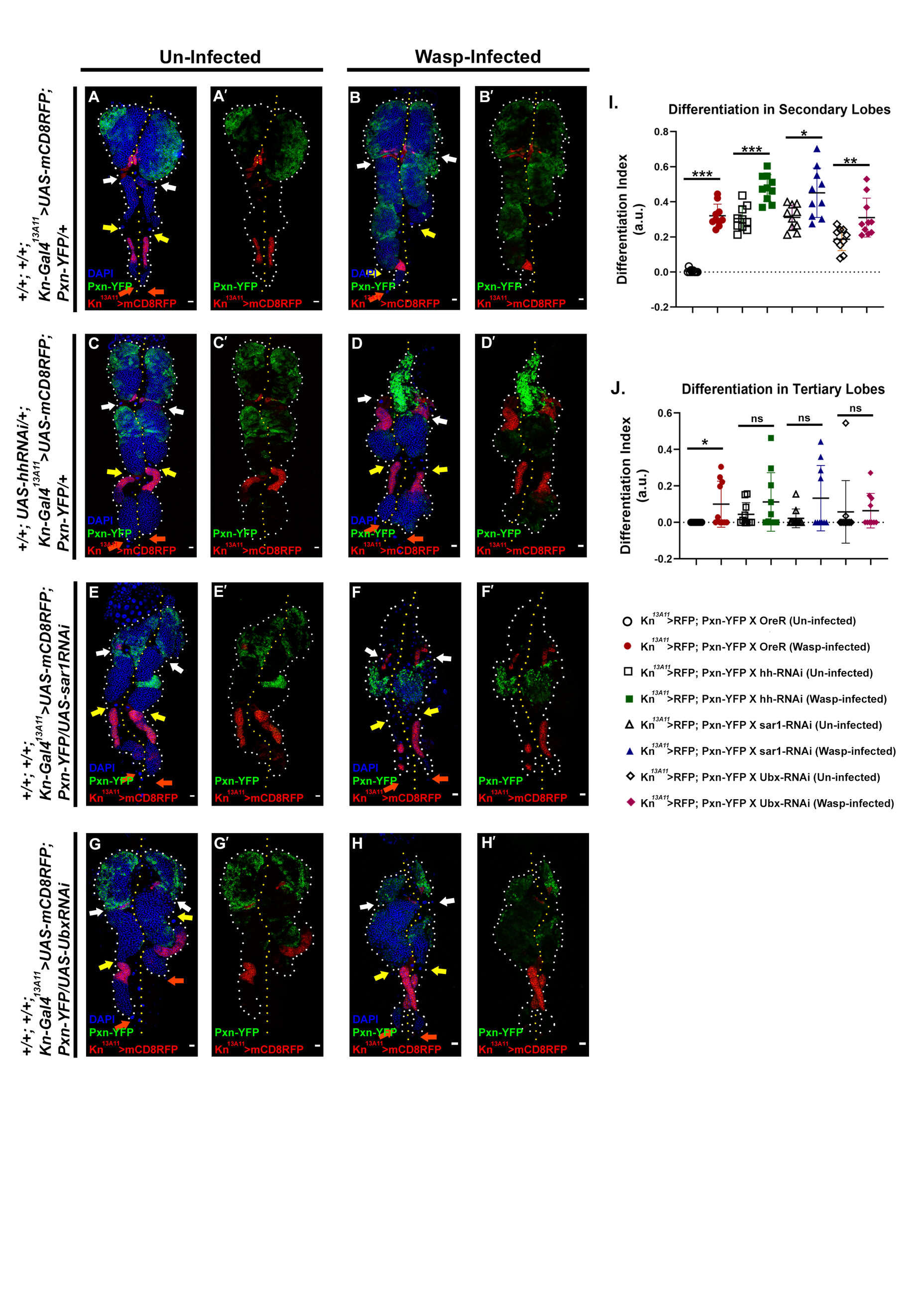

Supplement: S10 Fig — (A-H′.) Differentiation in response to wasp parasitism (B-B′) increases further when the functionality of the Ubx domain is perturbed via downregulation of hh (D-D′), sar1 (F-F′) and Ubx (H-H′) in comparison to their respective controls (A-A′), (C-C′), (E-E′) and (G-G′). (I.) Quantification of (A-H′) reveals the statistical significance of increase in differentiation of secondary lobes upon wasp infection. (Control: Un-infected vs Infected, n = 10, P-value = 6.886E-08; hh-RNAi: Un-infected vs Infected, n = 10, P-value = 3.947E-05; sar1-RNAi: Un-infected vs Infected, n = 10, P-value = 0.0144; Ubx-RNAi: Un-infected vs Infected, n = 10, P-value = 8.42E-03). (J.) Quantification of (A-H′) reveals the statistical significance of increase in differentiation of tertiary lobes upon wasp infection. (Control: Un-infected vs Infected, n = 10, P-value = 0.033; hh-RNAi: Un-infected vs Infected, n = 10, P-value = 0.237; sar1-RNAi: Un-infected vs Infected, n = 10, P-value = 0.088; Ubx-RNAi: Un-infected vs Infected, n = 10, P-value = 0.919). In all panels, lymph glands are dissected from third instar larvae (96hr after egg hatching, AEH). Arrows are pointed towards the intercalated pericardial cells within lymph gland lobes. The white arrow marks the pericardial cell between primary and secondary lobes; yellow between secondary and tertiary, and orange marks the last pericardial cell at the end of the LG organ after the tertiary lobe. White dotted lines mark the lymph gland boundary, while the yellow dotted line marks the aorta, which is between the bi-lobed lymph gland. The genotype of the larvae and labeling on the tissue is described in the panels. Scale bars: 20 μm. Two-tailed unpaired Student’s t-test was performed for calculating statistical significance. Data are mean±s.d. *P<0.05, **P<0.001 and ***P<0.0001. (TIF) [file pgen.1009709.s010.tif]

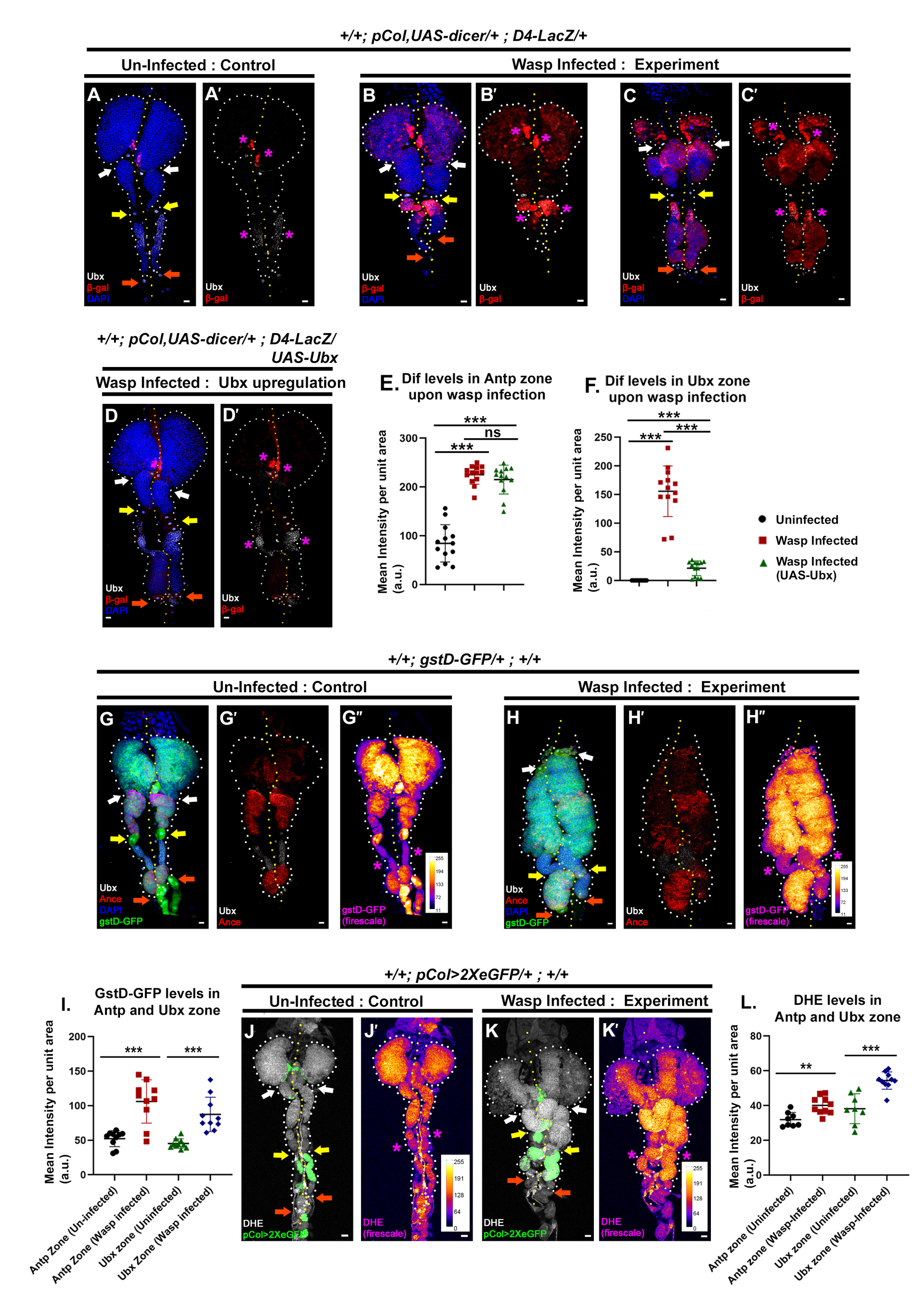

Supplement: S11 Fig — (A-D′.) D4-LacZ (Dif) significantly increases in Antp zone as well as Ubx zone (B-B′ (mild response), C-C′ (strong response)) in comparison to control upon wasp infection (A-A′). Upregulation of Ubx via pCol-Gal4 suppresses Dif levels during wasp infection (D-D′), indicated by magenta asterisk. (E.) Quantitative analysis of D4-LacZ levels in Antp zone (Un-infected vs Infected: n = 13, P-value = 6.86143E-10). The increase in intensity is unaffected even upon Ubx overexpression via pCol-Gal4 (Un-infected vs Ubx overexpression: n = 13, P-value = 1.431E-09) and (Infected vs Ubx overexpression: n = 13, P-value = 0.320). (F.) Wasp infection induces D4 (Dif) in Ubx domain (Un-infected vs Infected: n = 13, P-value = 2.54857E-08) and its levels are rescued upon Ubx overexpression (Un-infected vs Ubx overexpression n = 13, P-value = 6.643E-05) and (Infected vs Ubx overexpression: n = 13, P-value = 4.813E-08). (G-H′′.) Wasp infection induces gstD-GFP in Ubx zone (H-H′′), compared to control (G-G′′), marked by a magenta asterisk. (I.) Quantitation of G-H′′ (Antp domain: n = 10, P-value = 3.08E-04; Ubx domain: n = 10, P-value = 3.67E-04). (J-K′.) Wasp infection generates ROS (DHE, K-K′) in the Ubx domain compared to uninfected (J-J′), marked by a magenta asterisk. (L.) Quantitative analysis of J-K′ (Antp domain: n = 8 (Control), n = 10 (infected), P-value = 1.58E-03; Ubx domain: n = 8 (Control), n = 10 (infected), P-value = 6.77E-04). In all panels, lymph glands are dissected from third instar larvae (96hr after egg hatching, AEH). Arrows are pointed towards the intercalated pericardial cells within lymph gland lobes. The white arrow marks the pericardial cell between primary and secondary lobes; yellow between secondary and tertiary, and orange marks the last pericardial cell at the end of the LG organ after the tertiary lobe. White dotted lines mark the lymph gland boundary, while the yellow dotted line marks the aorta, which is between the bi-lobed lymph gland. The genotype o [file pgen.1009709.s011.tif]
