# Supplementary material for: Ubx-Collier signaling cascade maintains blood progenitors in the posterior lobes of the Drosophila larval lymph gland
Source: PLoS Genet. 2021 Aug 9;17(8):e1009709. doi: 10.1371/journal.pgen.1009709 (PMC8376192; doi:10.1371/journal.pgen.1009709)
Supplement: S1 Table — (DOCX) [file pgen.1009709.s012.docx]

**Ubx-Collier signaling cascade maintains blood progenitors in the**

**posterior lobes of the Drosophila larval lymph gland**

Aditya Kanwal^1^, Pranav Vijay Joshi^1^, Sudip Mandal^2^ and Lolitika Mandal^1^*

**S1 Table: Fly Stocks used for the current study.**

| **Fly Stock** | **Source** |
| --- | --- |
| *w^*^ P{w^+mC^=PTT-un1}ZCL1973* | Lynn Cooley |
| *P{Pvf2-lacZ.C}* | M.A. Yoo |
| *P{hhF4f-GFP}* | R. A. Schulz |
| *dad-nRFP/TM3,Sb* | M. Gonzalez-Gaitan |
| *domeMESO-GFP* | U. Banerjee |
| *P{PTT-un}vkgG00454* | U. Banerjee |
| *w*; UAS-RFP,UAS-FLP,Ubip63FRT-STOP-FRT-GFP/Cyo;+/+* | U. Banerjee |
| *P{Ser-lacZ.II-9.5}* | U. Banerjee |
| *w*; P{GAL4}col85,UAS-dicer/CyoAct-GFP; D4LacZ/D4LacZ* | A. Sharma |
| *p{gstD-GFP}II* | D. Bohmann |
| *P {GAL4} col85/Cyo* | M. Crozatier |
| *P{GawB}Ubx^Gal4-M1^* | E. Sanchez-Herrero |
| *OreR* | Bloomington Stock Center (#BL5) |
| *y[1]w[*]; P{w[+mC]=UAS-mCD8.mRFP.LG}10b* | Bloomington Stock Center (#BL27399) |
| *y1 w*; P{UAS-mCD8.mRFP.LG}18a* | Bloomington Stock Center (#BL27398) |
| *w[*]; P{w[+mC]=UAS-2xEGFP}AH2* | Bloomington Stock Center (#BL6874) |
| *w1118; P{y+t7.7P{w+mC=GMR13A11-*  *GAL4}attP2* | Bloomington Stock Center (#BL49248) |
| *y[1] sc[*] v[1] sev[21]; P{y[+t7.7] v[+t1.8]=TRiP.HMS01403}attP2* | Bloomington Stock Center (#BL34993) |
| *w[*]; P{w[+mC]=tubP-GAL80[ts]}20; TM2/TM6B, Tb[1]* | Bloomington Stock Center (#BL7019) |
| *y[1] sc[*] v[1] sev[21]; P{y[+t7.7] v[+t1.8]=TRiP.HMS00355}attP2/TM3, Sb[1]* | Bloomington Stock Center (#BL32364) |
| *P{KK108916}VIE-260B* | Vienna Drosophila Resource Center (#109454) |
| *y[*] w[*]; P{w[+mW.hs]=GawB}NP7379 / CyO, P{w[-]=UAS-lacZ.UW14}UW14* | KYOTO Stock Center (DGRC#105442) |
| *w[1118]; PBac{802.P.SVS-2}Pxn[CPTI003897]* | KYOTO Stock Center (DGRC#115452) |
| *y[*] w[*]; P{w[+mW.hs]=GawB}NP1162 / CyO, P{w[-]=UAS-lacZ.UW14}UW14* | KYOTO Stock Center (DGRC#103908) |
| *w*; kn^col-1^ / CyO; P{col5-cDNA.C} / TM6B* | KYOTO Stock Center (DGRC#109023) |
| *w[*]; P{w[+mW.hs]=GawB}frc[NP0297] / TM3, Ser[1]* | KYOTO Stock Center (DGRC#103581) |
| *Ubx[1] e[1] / TM3, Sb[1] Ser[1]* | KYOTO Stock Center (DGRC#107481) |
| *w[1]; P{w[+mC]=UAS-Ubx.Ia.C}36.2 / TM3, Ser[1]* | KYOTO Stock Center (DGRC#106118) |

**Following stocks were created for this study:**

***1.) w; TepIV-Gal4, UAS-mCD8RFP/ cyo; +/+***

*a.) y[*] w[*]; P{w[+mW.hs]=GawB}NP7379 / CyO, P{w[-]=UAS-lacZ.UW14}UW14*

*b.) y1 w*; P{UAS-mCD8.mRFP.LG}18a*

***2.) w; +/ +; Kn-Gal4, UAS-mCD8RFP/ TM6b, Tb.***

*a.) w1118; P{y+t7.7P{w+mC=GMR13A11-GAL4}attP2*

*b.) y[1] w[*]; P{w[+mC]=UAS-mCD8.mRFP.LG}10b*

***3.) w; pCol-Gal4, UAS-dicer/ cyo, Ubx1/ TM6b, Tb***

*a.) w*; P{GAL4}col85,UAS-dicer/CyoAct-GFP; D4LacZ/D4LacZ*

*b.) Ubx[1] e[1] / TM3, Sb[1] Ser[1]*

***4.) w; +/ +; Kn-Gal4, UAS-mCD8RFP, Pxn-YFP / TM6b, Tb***

*a.) w1118; P{y+t7.7P{w+mC=GMR13A11-GAL4}attP2*

*b.) y[1] w[*]; P{w[+mC]=UAS-mCD8.mRFP.LG}10b*

*c.) w[1118]; PBac{802.P.SVS-2}Pxn[CPTI003897]*

***5.) w; tubgal80^ts^/ tubgal80^ts^; Kn-Gal4, UAS-mCD8RFP, Pxn-YFP / TM6b, Tb***

*a.)* *w[*]; P{w[+mC]=tubP-GAL80[ts]}20; TM2/TM6B, Tb[1]*

*b.) w1118; P{y+t7.7P{w+mC=GMR13A11-GAL4}attP2*

*c.) y[1] w[*]; P{w[+mC]=UAS-mCD8.mRFP.LG}10b*

*d.) w[1118]; PBac{802.P.SVS-2}Pxn[CPTI003897]*

***6.) w; pCol85-Gal4, UAS-2XeGFP/ cyo; +/ +***

*a.)* *P {GAL4} col85/Cyo*

*b.) w[*]; P{w[+mC]=UAS-2xEGFP}AH2*

***7.) w; +/ tubgal80^ts^/ tubgal80^ts^; Ubx(M1)-Gal4, UAS-mCD8RFP/ TM6b, Tb.***

*a.) P{GawB}Ubx^Gal4-M1^*

*b.) y[1] w[*]; P{w[+mC]=UAS-mCD8.mRFP.LG}10b*

*c.) w[*]; P{w[+mC]=tubP-GAL80[ts]}20; TM2/TM6B, Tb[1]*
