# Supplementary material for: Ubx-Collier signaling cascade maintains blood progenitors in the posterior lobes of the Drosophila larval lymph gland
Source: PLoS Genet. 2021 Aug 9;17(8):e1009709. doi: 10.1371/journal.pgen.1009709 (PMC8376192; doi:10.1371/journal.pgen.1009709)
Supplement: Attachment — Submitted filename: Response to ReviewersKanwal et al..pdf [file pgen.1009709.s015.pdf]

Response to Reviewers:

Manuscript Number PGENETICS-D-20-01903

Reviewer #1: This manuscript characterized the cell heterogeneity in the posterior lobes of Drosophila lymph lode and found a Ubx+Ance- zone might function as a potential niche regulate the posterior lobe progenitors. While this general finding is interesting, I find that the characterization of the niche function somewhat weak and casual. Figure 2 and 6 seem to diverge from the major point and logic flow and I think they should be left as supplemental figures.

Thank you for taking the time to review our work, and we are happy to know that you found it interesting.

Regarding shifting of Figures 2 and 6 to supplementary, we beg to differ with the reviewer. The work mainly focuses on the specification and maintenance of the posterior lobe progenitors. Our comprehensive analysis pinpoints the pivotal role of Ubx and Collier in this process. Fig 2: deals with the expression pattern of Ubx and Collier throughout development to demonstrate how their dynamicity etches out the progenitor vs potential niche domain. Since this information is essential to appreciate the follow-up analysis on functionality, we would like to keep it as a main figure.

Regarding Figure 6, which is currently Figure 7 in the revised manuscript, we would like to draw your attention to that we have used a specific driver to mark all the posterior lobe cells in the final version. Lineage tracing with this driver clearly demonstrates that the posterior lobes contribute towards the formation of the hematopoietic hub. This new and remarkable observation solidifies our previous finding, and, therefore, we would like to keep it as the main figure.

Specific points are listed as follows:

1. Figure 1A: Please mark the anterior/primary lobe and posterior/secondary/tertiary lobes clearly in the schematic view.

Thanks for your input. We have made the necessary changes in the revised version of the manuscript.

2. Figure 1B: why is Trol staining in posterior lobes significantly different from the anterior lobe (absent from the cells in the center)? The staining looks that Trol is on the periphery of these lobes so it seems to say it is uniformly stained among the cells.

Sorry for the confusion. We understand your concern. In the revised manuscript, we have rephrased the statement as "Moreover, *terribly reduced optic lobe* (Trol; (25)), a heparin sulfate proteoglycan seems to wrap up the entire posterior lobes (Fig. 1B, 1B″, 1B‴)".
Please refer to Page No: 6 and Lines: 130-131 in the current version.

3. Figure 1C-F, why is the morphology of secondary and tertiary lobes differ a lot from 1B? The secondary lobes in these panels contain only one lobe while in Figure 1B it contains 2 lobes.

As mentioned in the manuscript and several previously published literature, due to unknown reasons, there is variation in the size of posterior lobes from one genotype to another (Jung et al., 2005, Lanot et al., 2001, Benmioun et al., 2015, Letourneau et al., 2016, Yu et al., 2018). This variation in size often gets reflected, especially in different genotypes.

As mentioned in the manuscript, lobe number is defined by the presence of pericardial cells in between. Since the Figure referred, the lymph gland lacks a pericardial cell intercalating in between; we have considered it a single lobe. Please note that a thin row of cells in continuity within the lobe is evident in the DAPI image of Fig 1B.

For your convenience, we have shown it below. Kindly note the asterisk denoting the continuity.

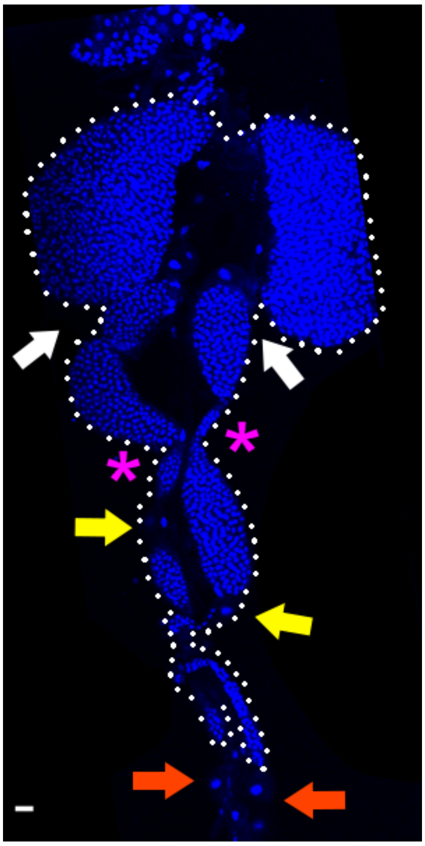

4. Figure 1H: Upon wasp infection, is there a shrinkage of the Ance- domain of the tertiary lobe? Moreover, I don't see why the data shown rule out the possibility Pxn+ in the Figure didn't rise from Ance- cells.

Kindly note that Ance is a pan progenitor marker (localizes with dome and TepIV) (Sharma et al., 2019). Pxn positive cells differentiate from the progenitors that are Col positive yet Ubx negative. This data is illustrated through our detailed analysis. Please refer to the S3 A-D''' Fig of the current version.

5. Figure 1I: The schematic Figure here seems completely unnecessary.

We disagree with this point and would like to draw the reviewer's attention to the fact that the progenitors in the posterior lobes were largely uncharacterized. Most of the studies referred to them as a homogeneous pool of progenitors.

Based on our expression studies, the results in the first panel illustrate that heterogeneity exists in the posterior lobes. We, therefore, have attempted to update the existing model (summarized in Figure1I)

6. Figure S1D: is it two domains? The Figure looks like three domains.

Sorry for the confusion. We understand the issue raised. However, all of our analyses reproducible indicated the existence of two domains. The artefact is sample-specific, created most likely by the twisting of the slender lobes while mounting. To remove the confusion, we have replaced it with a representative image in the revised version. Thanks for pointing this out.

7. Figure 1 title: Please rephrase "Posterior Lobes are a heterogeneous bunch of cells" to academic language.

We have rephrased it with the following statement: "Posterior lobes house heterogeneous cell populations" Please refer to Page No: 34 and Line No : 842.

8. In Figure 2, please explain how the arrows are drawn to define the secondary vs. tertiary lobe.

As accepted in the field, our reference point is the pericardial cells (indicated by coloured arrows) intercalated between the lymph gland lobes. We understand that the description is needed for all figure legend and have included that in the revised version.

9. In Figure S2, I find the choice of staging to 110h AEH incompatible with the staging in Figure 1, if the authors want to show that Ance- region previously describe in Figure 1 is Ubx+, use the same staging is essential.

As mentioned in the text, regression of Collier and Ubx though initiates early on but is most evident during 110h AEH. In the absence of markers that are limited to one vs the cell type, we felt the best stage to illustrate the regression phenomena is 110 AEH.

10. Figure 4A-J, Ubx and Pxn in the same channel is very difficult for readers to visualize in the Figure.

Since antibodies for Ubx and Pxn are raised in mice, we had faced this technical difficulty. However, we could interpret the data since Ubx is nuclear and Pxn is membranous. Moreover, in the current version, we have also analyzed loss of *Ubx* with Ubx(M1)-Gal4 and observed similar results. Please refer to Figure 5 of the revised manuscript.

11. Define how differentiation index is calculated.

Thanks for raising this issue. We have provided the details of our steps in calculating the Differentiation Index in the Material and Methods section of the revised manuscript. Please refer to page No: 24 and Lines: 574-585.

12. I don't understand what is sar1 and why downregulating it blocks niche signaling. Moreover, the result in Figure S4 seems important enough to be incorporated to Figure 4.

Newly synthesized proteins are transported from the endoplasmic reticulum to Golgi via coat protein complex II (COPII), the formation of which is regulated by Sar1. Thus, the knockdown of sar1 leads to the intracellular accumulation of proteins that are otherwise meant to be released extracellularly. Please note that downregulation of *sar1* has been previously employed in the lymph gland to block cell-cell communication (Destalminil-Letourneau, M., Morin-Poulard, I.,

Tian, Y., Vanzo, N. and Crozatier, M., 2021. The vascular niche controls Drosophila hematopoiesis via fibroblast growth factor signaling. *Elife*, *10*, p.e64672).

We have now included a short description in the text. Please refer to Page No:14 and Lines. 323-330.

Following reference on the function of Sar1 and its use in blocking cell-cell communication has also been incorporated.

Saito Y, Kimura K, Oka T, Nakano A. Activities of mutant Sar1 proteins in guanine nucleotide binding, GTP hydrolysis, and cell-free transport from the endoplasmic reticulum to the Golgi apparatus. J Biochem. 1998 Oct;124(4):816-23. doi: 10.1093/oxfordjournals.jbchem.a022185. PMID: 9756629.

Juan S Bonifacino an Benjamin S Glick, 2004 The Mechanisms of Vesicle Budding and Fusion. Cell 116, ISSUE 2, P153-166

Pastor-Pareja, J.C. and Xu, T., 2011. Shaping cells and organs in Drosophila by opposing roles of fat body-secreted Collagen IV and perlecan. *Developmental cell*, *21*(2), pp.245-256.

Saito, K., Maeda, M. and Katada, T., 2017. Regulation of the Sar1 GTPase cycle is necessary for large cargo secretion from the endoplasmic reticulum. *Frontiers in cell and developmental biology*, *5*, p.75.

Destalminil-Letourneau, M., Morin-Poulard, I., Tian, Y., Vanzo, N. and Crozatier, M., 2021. The vascular niche controls Drosophila hematopoiesis via fibroblast growth factor signaling. *Elife*, *10*, p.e64672

12. What is Figure 4K and L? There is no explanation in the article.

We want to draw your attention once again. It has been called and explained in the result section. We have elaborated on it in the revised version. Please refer to Page No: 13 and lines: 297-300.

13. Figure 5, what is the timing of this experiment? I find overexpression of Ubx in a domain where its expression is already high somewhat puzzling.

Experimental details have been mentioned in the main text (Line: 151, Page No: 7) and material method section (Lines: 553-559, Page No: 24).

For the experiment mentioned, we speculated was that Ubx over-expression from the Ubx domain could strengthen the niche functionality by boosting the maintenance signal, which then can help in rescuing the ectopic differentiation due to wasp parasitism. In the revised version of

our manuscript, we have demonstrated that Hh levels (transcriptional reporter: hhF4fGFP) increase upon Ubx over-expression, reflecting the increase in niche functionality. Please refer to S9 Fig and Lines: 371-373, Page No. 16 Main Text.

What is the phenotype upon infection when Ubx or hh is knocked down?

Thanks for your suggestions. We have performed these experiments. Our results show that wasp infection on the genotypes are critical for niche functionality (like loss of Hh or sar1 or Ubx from the PLSC) enhances the infection response assayed via differentiation index. We have included it in the revised version (S10 Figure and Lines: 375-381, Page No. 16 in the main text)

Minor point:

Line 97 “unique zonation signifying distinct cell types within these lobes” please rephrase for clarity.

We have rephrased this to “unique pattern of zonation signifying distinct cell types within these lobes” Please refer Page: 5 and Line: 109 of the revised manuscript.

Reviewer #2: Review uploaded as an attachment

Manuscript Number PGENETICS-D-20-01903

Comments for the authors

This manuscript by Kanwal et al., adresses a rather unexplored aspect of Drosophila larval hematopoiesis, the regulation and fate of cells in the so-called posterior lymph gland lobes, in normal development and upon wasp parasitization. The developmental expression patterns shown in each Figure beautifully document for the first time the posterior LG lobes in late larvae and early pupae.

Thank you very much for your appreciation. It means a lot to us.

The presented data convincingly allow to distinguish the progenitor status of the secondary and tertiary lobes and draw some new conclusions on the role of homeotic genes in controlling hematopoiesis in the Drosophila LG. They also expand the LG niche concept by drawing parallels between Col/Antp-expressing cells in primary lobes and Col/Ubx cells in secondary lobes. Altogether, this ms should represent a valuable contribution to our knowledge of Drosophila hematopoiesis. Drosophila is a reference genetic model for studying animal innate immunity and this paper should be of interest to a wide audience.

Thanks a lot. We are much encouraged by your valuable comments. Deeply appreciate your time and input in reviewing our manuscript.

However, before publication, important revisions are needed.

The conclusions of the authors are essentially based on expression/RNAi patterns obtained with one Gal4 driver. The present data need to be completed by a full description of this driver activity, compared to Col/Kn Antibody/ISH stainings. Morover, the author's conclusions require to be supported by additional experiments, using a driver not expressed in PSC cells (the « classical » niche).

Thanks for your guidance. To address your concerns, we have done the following experiments:

A. The present data need to be completed by a full description of this driver activity, compared to Col/Kn Antibody/ISH stainings.

The Kn/Col-Gal4 driver lines were previously described (Krzemien et al., 2007, Tokusumi et al., 2017, Carayon et al., 2020). However, we understand the concern and have done antibody labeling of Col to demonstrate the expression domains of the two Gal4 constructs used for our analysis.

Please refer to S2 Figure and Lines: 185-191, Page No.8 of Main text.

B. Morover, the author's conclusions require to be supported by additional experiments, using a driver not expressed in PSC cells (the « classical » niche).

Thanks for this input. We have used Ubx(M1)-Gal4 as an alternate driver to endorse our findings in the revised manuscript. Please note that the Ubx(M1) Gal4 is only expressed in the posterior lobes and not in the primary PSC. Please refer to the S5 figures. We have mentioned this in the Main text: Page: 13 and Lines: 301-315.

Ideas of the authors on the potential role of Col/Ubx "progenitor" cells traced in adults also need to be clarified, and a summary figure added. This is an mportant point, since, no functional data support the existence of adult hematopoiesis in Drosophila, notwithstanding the fact that the persistence of some hematopoietic progenitors in adults is itself subject to caution.

We have performed lineage tracing analysis with a posterior lobe specific Gal4 in the revised manuscript. Ubx(M1)-Gal4 on lineage tracing throughout development marks the posterior lobes. However, the live expression is limited to the anterior part of the tertiary lobe/PLSC co-localizing with Ubx antibody (please refer to the S5 Figure). We have summarized our previous and the recent result in Figure 7 of the revised manuscript.

Studies employing PH3 and BrdU have shown that hemocytes in the hematopoietic hub have the potential to proliferate upon bacterial challenges (Ghosh et al., 2015, Ghosh et al., 2018).

Fig. 2 : In this and subsequent figures, Knot-Gal4 is taken as reporter of Col/Knot expression. This simplification is triply misleading.

1) Formally, there is no valuable reason to call this driver Knot/kn-Gal4, since it is not an insertion in the locus. There are many drivers made from col/kn introns or upstream/downstream regions which have already been characterised. This one, GMR13A11. col/kn GMR lines has already been studied by other labs (Tokosumi et al ; 2017 ; Carayon et al., 2020), showing that GMR13A11 only reproduces part of the col :kn expression in the LG.

We agree with the reviewer; these papers indicate that GMR13A11 (Kn-Gal4) partially reproduces Col/Kn expression in LG. We chose Kn-Gal4 as we found it to be very specific for its expression in LG. Least expression was found in other tissues in comparison to pCol-Gal4. Moreover, the Kn-Gal4 and pCol-Gal4 time series with Col antibody in the recent version of this manuscript further endorses our decision to use Kn-Gal4, which seems to co-localize with the high Col antibody cells, which we wanted to address in our study. Please refer to the S2 Figure in the revised version.

2) To conclude on the dynamics of col/kn expression during LG maturation requires Col/Kn antibody staining, since Col/Kn antibodies are available.

Thanks for your suggestion. We have now included the Kn-Gal4 and pCol-Gal4 time series with Col antibody in the recent version of this manuscript. Please refer to the S2 Figure and Lines: 185-191, Page No.8 of Main text in the revised version.

3) Unlike Col/Kn, GMR 13A11 is not expressed in primary lobe progenitors but strongly expressed in the PSC. A key conclusion of the paper, namely that the anterior tertiary lobe acts in a non cell-autonomous manner (as a niche) for maintaining secondary lobe progenitors, is based on one experiment using a similar driver (pCol-Gal4/Ubx RNAi experiment; see also comments on Fig. 4). This conclusion needs to be confirmed using a driver not expressed in the PSC. In other terms, that the PSC does not act as a niche for secondary lobe progenitors needs to be ruled out. What is crucially missing is a confirmation of the author's conclusions by repeating experiments using a driver expressed in posterior lobes but NOT EXPRESSED in PSC cells, unlike GMR13A11 or Pcol85.

As mentioned earlier, UbxM1-Gal4 only expresses in the posterior lobes and is totally absent in the primary lobe (S5 Figure). Using this Gal4, we have carried out the functional assays to address the reviewer's concern.

In order to assess the role of primary lobe PSC in the maintenance of posterior lobe progenitors, we knocked down *hh* and *sar1* from the PSC and assayed differentiation in the posterior lobes. Intriguingly, we do see a few cells differentiating in the posterior lobes (please see Figure below). Based on this observation, we can infer that the primary lobe PSC does have a partial contribution to the maintenance of posterior lobes.

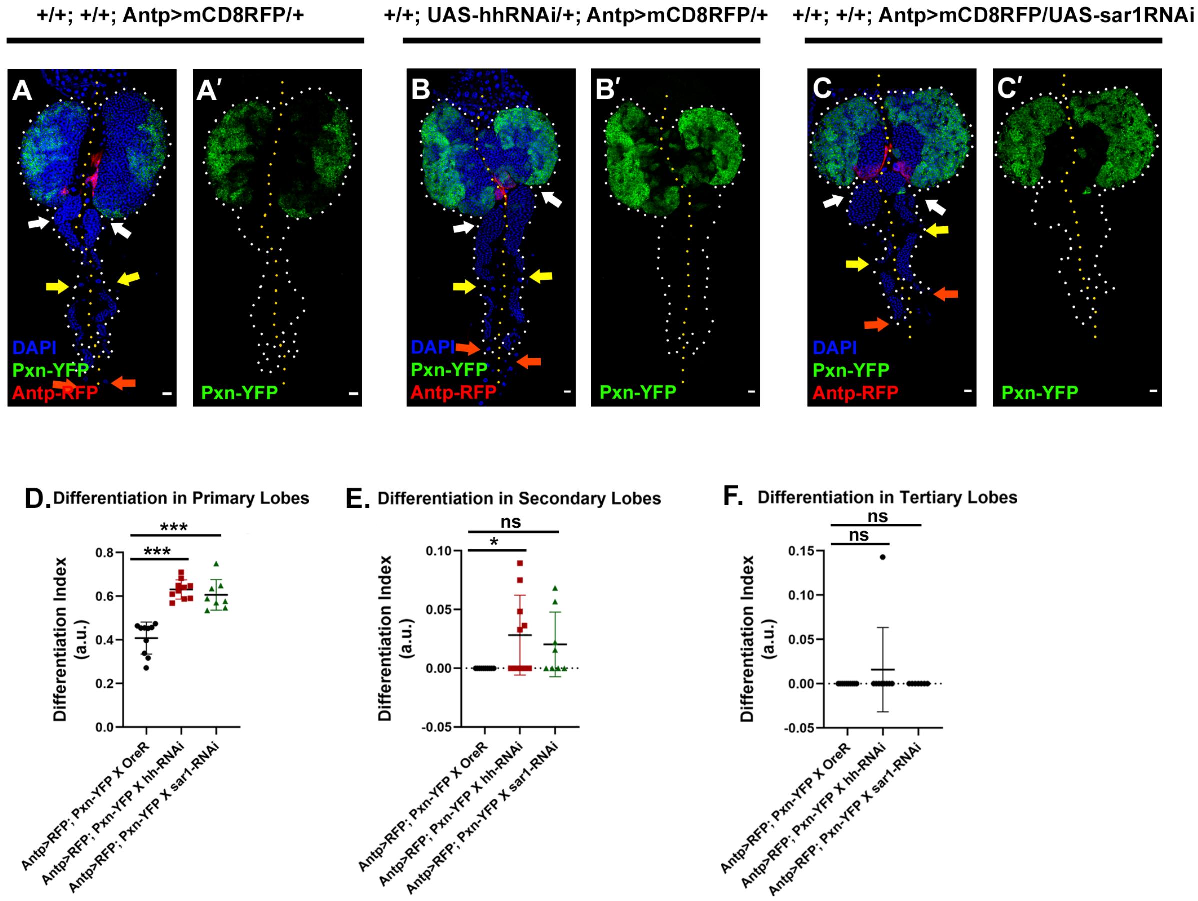

Fig 4, 5 and S5: What is crucially missing is a confirmation of the author's conclusions by repeating experiments using a driver expressed in posterior lobes but NOT EXPRESSED in PSC cells, unlike GMR13A11 or Pcol85.

Please refer to our response above. We have repeated all the experiments with posterior lobe specific driver, which is not expressed in the PSC. However due genetic constraint, we were unable to repeat the experiment shown in S5 (which is S11 of the current manuscript). To perform this experiment with posterior lobe specific driver :Ubx(M1)-Gal4 which is on third chromosome was technically difficult, since both D4-Lacz and UAS-Ubx are also on third

chromosme. Except this one experiment rest have beenrepeated with the posterior lobe specific driver.

Fig. 4

4C-E ; Pxn expression reveals hemocyte differentiation in the secondary lobes. In E and possibly C, there is an anterior Pxn cluster, close to the PSC. How do the authors interpret this cluster ?

The Pxn positive cluster is at the different stack of the Z section of the confocal image. Because the merging has been done for image processing, it appears close to PSC.

pCol(85), another driver than GMR 13A11 is specifically used for Fig. 4A-F. Why ? Why using two different drivers in separate experiments. At minimum, the relative dynamics of GMR13A11 and pCol expression in the tertiary lobes should be compared, and compared to that of Col/Kn It is essential to add Col antibody stainings to panels A-E

Both Ubx^1 null allele and Knot-Gal4 are on the 3rd chromosome. Due to this genetic constraint, we used pCol85-Gal4 on third for this specific experiment. However, in the revised version of the manuscript, to further provide additional evidence to our findings, we have used an independent Gal4: Ubx(M1)-Gal4.

Please note that the relative expression dynamics of these two drivers of Collier/Knot have also been included in the revised version of the manuscript S2 Figure.

We found in both cases a significant concomitant drop in Col and Ubx protein levels, assayed by immunofluorescence studies. Please see Figure 5, where Ubx(M1)-Gal4 is utilized in the revised version of the manuscript and Figures below, where pCol-Gal4; Ubx^1 is employed. Thanks for your suggestion. It has strongly endorsed our observations.

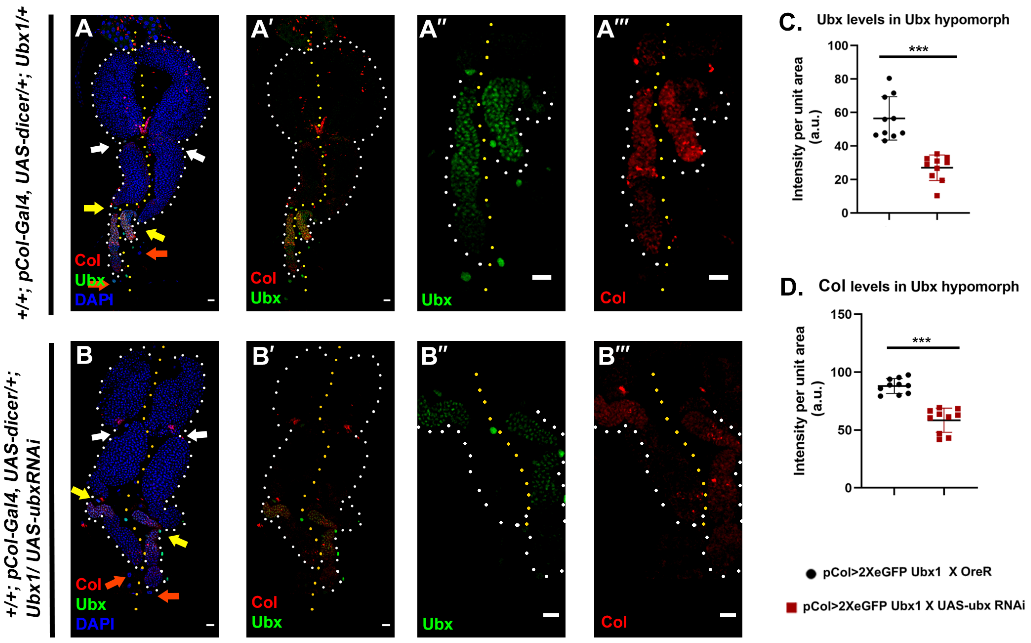

Ubx and sar1/hh (sup fig.4) loss of function reveal a non autonomous (niche) role of Ubx on progenitors in secondary lobes. Ectopic Ubx gain of function in secondary lobes leads to conclude to a cell-autonomous role in preserving progenitors. Could the authors discuss this apparent contradiction !

We beg to differ with the reviewer in this context. This is not an issue of contradiction. Upon the loss of *Ubx*/ *hh*/ *sar1* from the Ubx domain of the posterior lobe affects the progenitor state and pushes them toward differentiation. Likewise, upregulating Ubx from the same domain empowers the niche to increase the maintenance of the progenitors. To confirm our hypothesis of strengthening the niche functionality, we assayed levels of Hh (using the transcriptional reporter hhF4fGFP). Upon upregulation of Ubx, we see a significant increase in Hh levels, thereby indicating an increase in niche functionality. Please refer to S9 Figure and Lines: 371-373 and Page 16 in the main text. We would also like to point out that the loss of Ubx from this domain results in compromised Hh expression, affecting progenitor maintenance (please refer to the Figure below).

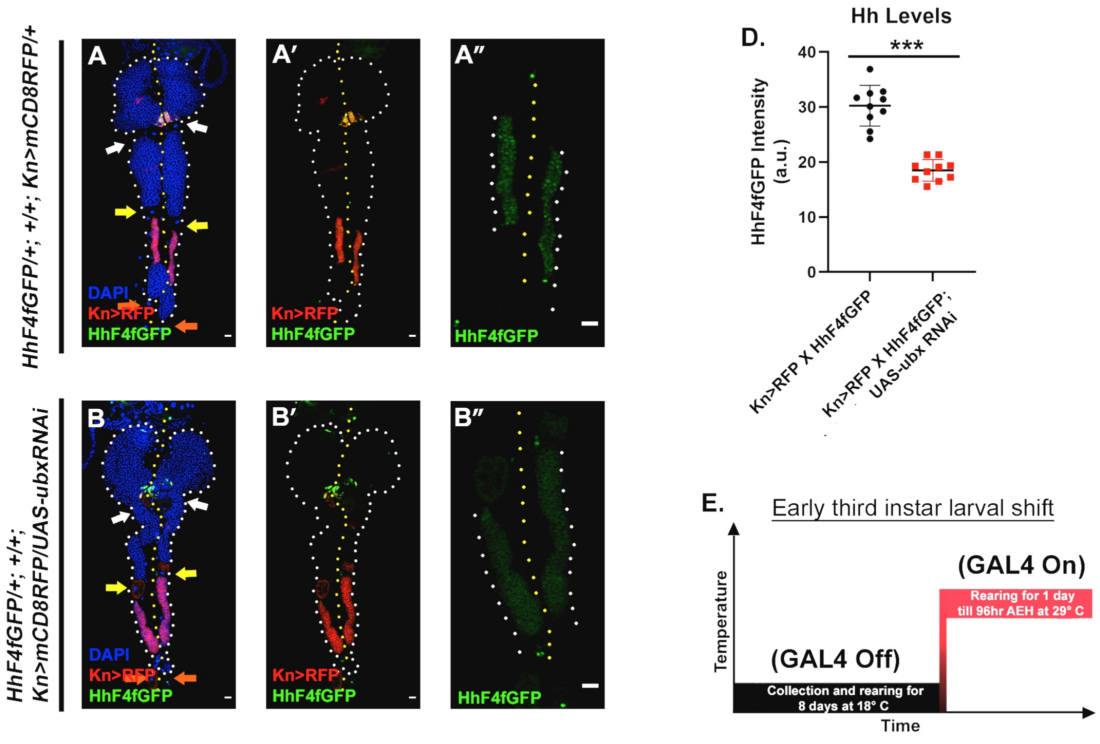

The number of « Kn/Col expressing cells is also very increased in secondary lobes upon ectopic Ubx expression. Could the authors comment on this.

Based on our epistatic analysis, Col is downstream of Ubx, just like the primary lobe where it is downstream to Antp. Previous studies have demonstrated how Collier is essential for maintaining the niche cell numbers (Pennetier et al., 2012). Although it needs to be addressed but based on the epistatic analysis, we speculate a similar function of Col in the Ubx domain of the posterior lobes.

Fig. 5S D-F. Do the Ubx levels significantly drop upon infestation ? Add a secondary lobe specific driver

Following the reviewers' suggestion, we have used a posterior lobe-specific driver Ubx(M1)-Gal4 for this experiment. Please refer to S7 Figure.

As suggested by the reviewer, Ubx levels were assessed upon wasp infection. We employed Pxn-YFP so that we can visualize the differentiation status upon wasp parasitism. A significant drop in the levels of Ubx in posterior lobes was evident upon immune challenge (S8 Figure).

Fig.6 The title and paragraph, lines 354-381 is very confusing. Does the late-restricted Ubx/Col expression domain correspond to a « niche » or to a « discrete progenitor pool », lines 358.

My understanding of the cell-tracing experiments is that these cells are niche cells, not or rarely found in adult hematopoietic hubs, while some progenitors expressing Col earlier might contribute to adult hemocytes. They express progenitor markers in early pupae, but what about in adults ? To my best knowledge, there is no evidence for mitotically active hematopoietic cells in adults (Sanchez Bosch et al., 2019). BrdU experiments (Ghosh et al., 2015, 2018) did not rule out endocycling of post-mitotic cells. The new experiments by Kanwal et al., raise the question of the role of the late-traced cells in adults. This point should be considered in the discussion.

Although Col is widespread in the posterior lobes, co-expression studies with Ubx reveal that it with development, it gets restricted to the Ubx domain. This restriction defines two domains: one, the potential niche, which is high in Ubx and Col expression, while the other Ance+, Ubx- and Col^{Low} enriched domain represents the progenitor cells. Our lineage-tracing experiments were performed at different time windows that were selected based on the restriction of Col. We show that upon activation of the LT cassette by Kn-Gal4 at 72 hrs, the number of lineage traced cells were pretty high in the hematopoietic hubs. However, with time and with restriction of Col towards the Ubx domain/PLSC led to a decline in the number of lineage traced cells that populate the hub. This clearly indicates that very few Col+ progenitors are left at 120 hrs that gets lineage trace to the hub. We firmly believe that this Col lineage traced cells are not the niche cells as they are majorly P1 positive in the adult hub.

Thanks for drawing our attention towards this aspect. We have now mentioned this in the main text (Page: 18, Lines: 420-424).

As far as mitotically active hematopoietic cells in the hub are concerned, we want to draw your attention towards a publication from our group which demonstrates the presence of PH3 positive hemocytes post bacterial challenge (Ghosh S, Mandal S, Mandal L. Detecting proliferation of adult hemocytes in *Drosophila* by BrdU incorporation and PH3 expression in response to bacterial infection. Wellcome Open Research. 2018;3.)

Figure 6 itself is confusing

Fig.6 itself. Panel A is particularly confusing. Non only should it be placed after the experimental data panels bu also explained. The adult « fate » of the secondary lobe and posterior tertiary lobe cells should be distinguished from the fate of the Kn/Ubx anterior cells of the tertiary lobes.

Figure 6 currently is Figure 7 of the revised manuscript and has been modified with recent findings. Keeping the suggestion in mind, we have modified the panel. We have incorporated our recent lineage tracing experiments with posterior lobe specific driver Ubx(M1)-Gal4. Continuous activation of the driver demonstrates that the hematopoietic hub harbours many Ubx lineage traced cells. A Majority of them are P1 positive. This result solidifies our previous observation that, indeed, posterior lobes contribute toward the hematopoietic hub.

We have summarised it as an infographic represntation in Figure 7D. In this panel, we have also incorporated the results of lineage tracing Kn-Gal4 at different time points to reveal the contribution of Col positive cells towards the hematopoietic hub. The decline in the number of lineage traced cells indicates that by 120hr AEH, the number of Col+ progenitors has dwindled. Interestingly most of the cells are P1 positive thereby indicating that they are derived from the Col expressing progenitors of the psoterior lobes (Figure 7E-K).

A summary figure of the new findings is absolutely necessary.
Thanks for your suggestion. A Summary model has been included (Fig 8).

Minor comments ; suggestions for improvement.

Introduction

The authors cite Rodrigues et al, 2020 who also study secondary and tertiary lobe progenitors in pre-pupae, based on expression patterns. There is some discrepancy between Rodrigues and the submitted article on the expression patterns of HhGal4 and SerGal4. Please clarify this. I would ne nicely complemented by looking at GMRA11>Ubx-RNAi LGs.

Ser reporter lines used by Rodrigues et al., 2020, and this study are different. They have used Ser-Gal4, whereas we have employed Ser 9.5 LacZ (Lebestky et al., 2003). This might have led to the difference in observation.

In our study, we have used the first chromosome hhF4fGFP construct, and we are not sure which specific reporter of hhF4fGFP is utilized in their study. Moreover, as shown in our study, the hhF4fGFP levels in the Ubx domain are low compared to those in the Antp domain. This might be one of the reasons why it missed their attention.

Thanks for the suggestion; we assayed Hh levels in flies where *Ubx* was knocked down from PLSC/Ubx domain. We found a substantial drop in Hh levels in such genotypes, further asserting the function of Ubx upstream of Hh in posterior lobes.

The images are provided on page 12 of this document.

Compartimentalisation of the niche (PSC) by ECM proteins was first documented by Krzemien et al., 2010. This and the compartimentalisation of the tertiary and secondary lobes raise the question of the mode of communication between ECM-separated groups of cells,a topic which could possibly be underlined in the discussion.

Thanks for your suggestion. This thought has been incorporated in the Main text. Please refer to Line: 278-280 and Page: 12.

Results.

All figures

Indicating Pxn, Kn/Col, Dad etc, in red on the figure panels is misleading. What is detected is Pxn-YFP and GMR13A11>mCD8GFP, etc, expression, not the endogenous protein expression. For other reporters, the labelling is correct. Please chek all figures for consistency.

Thanks for pointing it out. All the changes have been made in the current version of the manuscript.

Fig2S. What is the colPS mutation which is not listed among fly stocks? Is it col1 ? In this case please refer to is as col1.

Thanks for drawing our attention. Yes, it is col^{1} (Genotype: *w*; kn*$^{col-1}$ */ CyO; P{col5-cDNA.C}/TM6B*). We have made these changes in the current version.

2S E-J': ColPS posterior lobes F , I, J are much larger than wild type; please comment.

This is due to excessive differentiation (as observed by the P1) in col loss of function. To represent the variation often encounter in the otherwise highly differentiated lymph glands of *col*^{1}, we have included two examples in the same panel (S3 F-G' Fig).

In G, the tertiary lobe is drawn without evidence from DAPI staining ?

Sorry for the confusion created. We have re-processed the same image to clear the confusion

Fig. S3 The level of Ser expression in tertiary lobes seems more similar to Ser expression in primary lobes outside the PSC in early third instar (Crozatier et al., 2004 ; Spratford et al., BioRxiv, 2020) than in the PSC. Unlike frc, Ser and Lamin-C are expressed in the entire tertiary lobes, and not restricted to the posterior domain.

As mentioned in the text, at 96hr AEH the expression of these markers include a subset of Ubx positive cells.

Fig. 4E There is a cluster of Pxn positive cells in the posterior tertiary lobe. It would be better to show a detail of this entire lobe in F.

A zoomed-in image including the Pxn positive cells has been included in the current version of the manuscript as suggested by the reviewer (Figure 4G-G').

Fig. S5, lines347-349 : In contrast, increased gstD-GFP levels were observed in the Ubx domain upon wasp infection, in a manner identical to that of Antp expressing PSC/niche (Fig. 5S K-K′, L). The level of gstD remains low in Ubx cells upon wasp infestation, compared to other cells and it is not possible to compare with Antp cells on this Figure.

Quantification of gstDGFP and DHE in Ubx vs. Antp domains in the normal scenario and upon infection has been provided in the new version of the manuscript, in order to endorse our claim. (please refer to S11 I, S11 L Figs)

Reviewer #3: The lymph gland of Drosophila plays a significant regulatory role in larval hematopoiesis. Several functional cellular compartments and regulatory factors have been identified, however the studies focused mainly on the anterior lobes of the organ. Developmental studies suggested that the posterior lobes and their regulation is different from those of the primary lobes, but their regulation, their role in hematopoiesis and in the immune response has not been explored. The Authors of the presented studies reveal the functional heterogeneity of hematopoietic progenitors residing in these lobes and identify regulatory factors acting in the separate niches and responsible for the regulation of the distinct functional progenitor populations.

Thanks a lot for your time and comments. They are deeply appreciated.

Minor comments:

Lines 65 and 691: SHRESTHA and GATEFF are in capital

Referencing was performed using EndNote software, which automatically types SHRESTHA and GATEFF reference in capital.

Cited articles missing from "References" section:
line 465 Kurucz et al., 2007
line 467 Shirras , Hurst et al., 2003

Thanks for pointing it out. We have included these references in the current version of the manuscript. Please refer to Page: 33 and Lines: 831-836 of the revised manuscript.

Have all data underlying the figures and results presented in the manuscript been provided?

Large-scale datasets should be made available via a public repository as described in the *PLOS Genetics* data availability policy, and numerical data that underlies graphs or summary statistics should be provided in spreadsheet form as supporting information.

Reviewer #1: Yes

Reviewer #2: Yes

Reviewer #3: Yes

PLOS authors have the option to publish the peer review history of their article (what does this mean?). If published, this will include your full peer review and any attached files.

If you choose "no", your identity will remain anonymous but your review may still be made public.

Do you want your identity to be public for this peer review? For information about this choice, including consent withdrawal, please see our Privacy Policy.

Reviewer #1: No

Reviewer #2: **Yes:** Alain VINCENT

Reviewer #3: No
