# Supplementary material for: Ubx-Collier signaling cascade maintains blood progenitors in the posterior lobes of the Drosophila larval lymph gland
Source: PLoS Genet. 2021 Aug 9;17(8):e1009709. doi: 10.1371/journal.pgen.1009709 (PMC8376192; doi:10.1371/journal.pgen.1009709)
Supplement: Attachment — Submitted filename: Response to Reviewers 28-06-21.pdf [file pgen.1009709.s016.pdf]

Response to Reviewers' Comments

Title: "Ubx-Collier signaling cascade maintains blood progenitors in the posterior lobes of the Drosophila larval lymph gland." **Manuscript Number PGENETICS-D-20-01903R1**

We thanks all the Reviewers for their time and input in enriching our manuscript.

Reviewer #1: While the manuscript has been improved, I am not yet convinced that the newly-identified Ubx zone functions as a signaling center. The functional assays of hh RNAi and sar1 RNAi using Ubx-Gal4 in this zone, provided in supplemental figure 6 and 10 seem to show rather minor and puzzling effects.

Thanks for your comments. We want to point out that all the phenotypes obtained after knocking down *hh* and *sar1* using Ubx(M1)-Gal4 (referred to as Ubx-Gal4^{M1} in the current version) mimics the phenotypes observed when Kn-Gal4 was utilized for the same.

We want to draw the Reviewer's attention to the fact that Kn-Gal4 is expressed in PSC and PLSC of the lymph gland (please refer to Figure 2 of the current manuscript). While Ubx(M1)-Gal4 expresses only in PLSC: please refer to Figure S5.Thus, the resulting phenotype is much more prominent in the case of Kn-Gal4 than the one obtained upon manipulating the PLSC alone by Ubx-M1 Gal4. The enhanced phenotype (differentiation defects) with Kn Gal4 can be attributed to the additive effect caused by disturbing the function of both the niches simultaneously.

In supplemental figure 6, loss of hh lead to increase of differentiation in secondary and tertiary lobes according to the authors. Why would loss of hh upon wasp infection lead to decrease of differentiation in the secondary and teritary zone.

Please note that there is an enhancement of differentiation defect upon simultaneously downregulating *hh* from the Ubx domain and infecting the same genotype with the wasp. This is pointed out and explained in **lines 375-378** of the main text and is evident upon quantitation in Figure S10. The secondary lobes of such larvae might seem smaller in size because many of the hemocytes are precociously released into

circulation due to this extreme insult. We have mentioned it in the revised version (**Page: 16 Lines: 377-378**).

We beg to disagree with the Reviewer's comment that "loss of hh upon wasp infection lead to decrease of differentiation in the secondary and teritary zone". Instead, we have written, "Blocking the communication between progenitors and the PLSC either by downregulation of sar1, hh or Ubx resulted in enhanced immune response upon Wasp challenge". Please refer to page No: 16 and Line: 375-378.

What is the phenotype of Hh over expression in the Ubx zone?

Although Hh overexpression was not done in the previous version, we had overexpressed Ubx, which resulted in elevated Hh expression in the PLSC. We would request the reviewer to note that the elevated Hh expression, in turn, resulted in increased maintenance of the posterior lobe progenitors (please refer to Figure S9).

We appreciate your input and have infected the individuals where Hh was overexpressed from the PLSC. Our result is analogous to Ubx overexpression; the upregulated Hh decreases differentiation compared to sibling control post-infection.

Please refer to figure below:

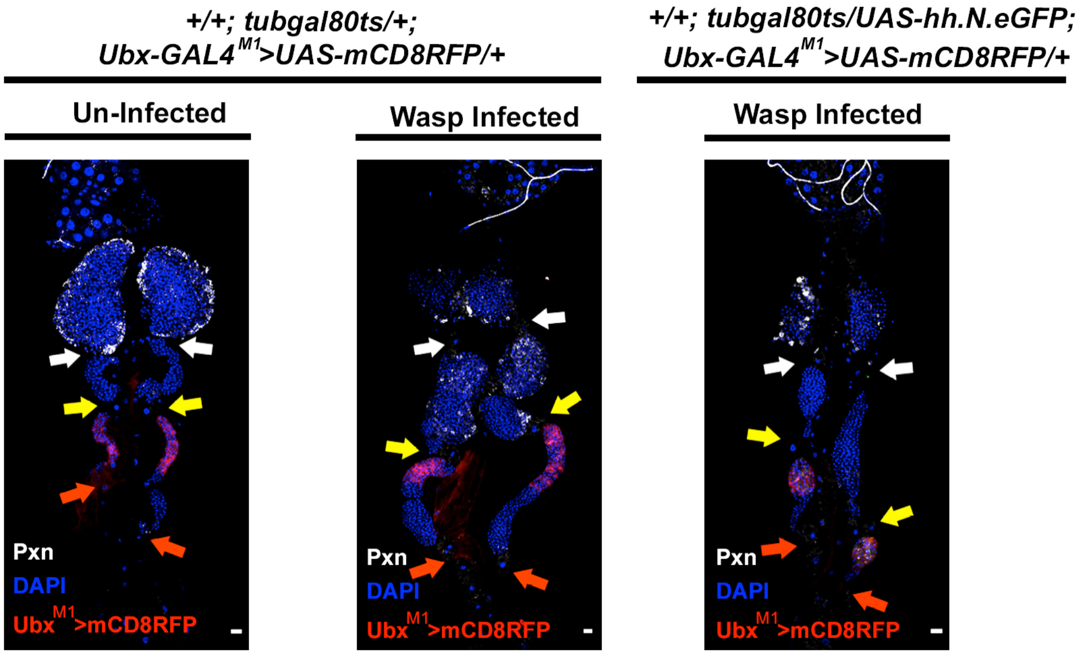

Figure Legend:

A. Lymph gland from uninfected control with PLSC marked in Red. Differentiation (white) can be seen only in the primary lobe.

B. Lymph gland from Wasp infected samples. The primary lobe is dispersed and is thus small compared to control. Differentiation is set in the posterior lobes.

C. Over expression of Hh in the PLSC prevents posterior lobe progenitors from differentiating. The primary lobe is largely dispersed due to differentiation and subsequent dispersal of the hemocytes.

Reviewer#2:

The revised manuscript by Kanwal et al., has satisfactorily answered most of my suggestions and criticisms. I than the authors for this. I feel that the revisions made have significantly improved their ms. Considering some main points I previously raised, revisions include : (1) A comparison between the two col/kn drivers which revealed a different temporality of expression (Fig.S2) ; (3) most importantly, an extension of the lineage and RNAi KD experiments using a driver expressed in secondary lobes but not the PSC (Fig. 4 and Fig.S5) ; these experiments nicely confirm the previous main conclusions of the authors, while revealing that the PSC contributes to the maintenance of progenitors in posterior lobes, a conclusion not reached in the initial version.

Thank you so much for your kind and encouraging remarks. Your suggestions and inputs have indeed strengthened the manuscript. Deeply apprecaite your input.

Before publication, a few more corrrections are needed.

Major.

Point 2, my concern about the KnGal4 nomenclature was not adressed. I would prefer not to compromise on this point. As I already wrote, the authors improperly use Kn-Gal4 to design a specific KnGal4 GMR line, line 13A11, characterised under this name to be expressed in the LG (Tokusimi et al., 2017). Many Kn/col enhancer and driver lines have previously been characterised, starting by wing enhancers (incidentally under Ubx control; Hersh and Carroll, Development 2005 PMID 15753212). I strongly suggest to refer to the specific KnGal4 line used by autors by its original name, GMR13A11 or KnGal413A11 (13A11 superscript). This may seem a detail but connecting the present data to previous data obtained in other labs and exploited here, would be fair and not confusing.

Thanks for your input.
We have replaced Kn-Gal4 nomenclature with Kn^{13A11}-Gal4 in the current version of manuscript.

Minor
- Contribution, both of the PSC and PSLC to maintenance of secondary lobe progenitors should be better emphasized in the discussion section, with a more moderate conclusion, line 465.

We have rephrased the conclusion and made relevant changes in the revised manuscript **(Page: 19-20, Lines: 465-475).**

- Fig.S1 A-C. there is a discrepancy/confusion between the text of legend S1 A-H' : Trol marks the progenitors in the primary lobe ; Viking expression marks the periphery of the primary lobe and the main text lines lines 257-264 . Similar enrichment of Trol in the niche of the primary lobes (25). Viking surrounding of the primary niche was first noted by Krzemien et al, 2010 (11) and Trol surrounding (and not enrichement) by Grigorian et al., 2013 (25). Please try to homogenise.

We have incorporated these suggestions in the current version of manuscript both in text and figure legend **(Page: 11, Line: 258 and Page: 47, Line: 1106-1110).**

-Fig 2B-B" text line 199 Kn should be KnGal4(13A11)
We have made this replacement in the current version of manuscript.

-Fig.4 J-J' Not only Ubx, but also Ubx(M1)-Gal4 seems down-regulated ; Does this reflect Ubx autoregulation of Ubx(M1) ? Please comment, if necessary.
We believe the reviewer is referring to Fig.5 J-J'. Indeed Ubx(M1)-Gal4 (referred to as $Ubx\text{-}Gal4^{M1}$ in the current version) expression is reduced when *Ubx* RNAi is performed from this domain. According to Navas et al., 2006, this Gal4 insertion (Position: 242,802) is very close to Ubx locus (from 242,867 to 320,921; Martin et al., 1995) and is "weakly mutant for the bithorax function", indicating that this Gal4 has a mild effect on Ubx function. We think this might explain the vice versa effect of knocking down *Ubx* over Ubx(M1)-Gal4 expression. Please refer to the following References:

1. Navas et al., (2006): A simple and efficient method to identify replacements of P-lacZ by P-Gal4 lines allows obtaining Gal4 insertions in the bithorax complex of *Drosophila*

2. Martin er al., (1995): Complete sequence of the bithorax complex of *Drosophila*.

Text: Check whether the phrasing is accurate for : lines 214-219 ; 210-212, Ux ?,Col ? regression or regression of both ?; 334-336 (Since ?) and 338 ; 360-363 ; 421-424 ; 467-468.

We understand your concerns and have edited the text accordingly. The changes are highlighted in the marked copy.

Lines 445-449 should be moved to discussion.

We have moved it to the discussion section **(Page No: 19-20, Lines: 465-475)** of the revised version.

Reviewer #3: This is an improved version of the manuscript.

Thank you for your kind remarks.
