# Supplementary material for: Ubx-Collier signaling cascade maintains blood progenitors in the posterior lobes of the Drosophila larval lymph gland
Source: PLoS Genet. 2021 Aug 9;17(8):e1009709. doi: 10.1371/journal.pgen.1009709 (PMC8376192; doi:10.1371/journal.pgen.1009709)
Supplement: Attachment — Submitted filename: Review of Kanwal et al, PGENETICS-D-20-01903.docx [file pgen.1009709.s014.docx]

Manuscript Number PGENETICS-D-20-01903

Comments for the authors

This manuscript by Kanwal et al., adresses a rather unexplored aspect of Drosophila larval hematopoiesis, the regulation and fate of cells in the so-called posterior lymph gland lobes, in normal development and upon wasp parasitization. The developmental expression patterns shown in each figure beautifully document for the first time the posterior LG lobes in late larvae and early pupae. The presented data convincingly allow to distinguish the progenitor status of the secondary and tertiary lobes and draw some new conclusions on the role of homeotic genes in controlling hematopoiesis in the Drosophila LG. They also expand the LG niche concept by drawing parallels between Col/Antp-expressing cells in primary lobes and Col/Ubx cells in secondary lobes. Altogether, this ms should represent a valuable contribution to our knowledge of Drosophila hematopoiesis. Drosophila is a reference genetic model for studying animal innate immunity and this paper should be of interest to a wide audience.

However, before publication, important revisions are needed. The conclusions of the authors are essentially based on expression/RNAi patterns obtained with one Gal4 driver. The present data need to be completed by a full description of this driver activity, compared to Col/Kn Antibody/ISH stainings. Morover, the author's conclusions require to be supported by additional experiments, using a driver not expressed in PSC cells (the « classical » niche). Ideas of the authors on the potential role of Col/Ubx "progenitor" cells traced in adults also need to be clarified, and a summary figure added. This is an mportant point, since, no functional data support the existence of adult hematopoiesis in Drosophila, notwithstanding the fact that the persistence of some hematopoietic progenitors in adults is itself subject to caution.

Major points to be addressed:

Fig. 2 : In this and subsequent figures, Knot-Gal4 is taken as reporter of Col/Knot expression. This simplification is triply misleading.

1) Formally, there is no valuable reason to call this driver Knot/kn-Gal4, since it is not an insertion in the locus. There are many drivers made from col/kn introns or upstream/downstream regions which have already been characterised. This one, GMR13A11. col/kn GMR lines has already been studied by other labs (Tokosumi et al ; 2017 ; Carayon et al., 2020), showing that GMR13A11 only reproduces part of the col :kn expression in the LG.

2) To conclude on the dynamics of col/kn expression during LG maturation requires Col/Kn antibody staining, since Col/Kn antibodies are available.

3) Unlike Col/Kn, GMR 13A11 is not expressed in primary lobe progenitors but strongly expressed in the PSC. A key conclusion of the paper, namely that the anterior tertiary lobe acts in a non cell-autonomous manner (as a niche) for maintaining secondary lobe progenitors, is based on one experiment using a similar driver (pCol-Gal4/Ubx RNAi experiment ; see also comments on Fig. 4). This conclusion needs to be confirmed using a driver not expressed in the PSC. In other terms, that the PSC does not act as a niche for secondary lobe progenitors needs to be ruled out.

Fig 4, 5 and S5

What is crucially missing is a confirmation of the author’s conclusions by repeating experiments using a driver expressed in posterior lobes but NOT EXPRESSED in PSC cells, unlike GMR13A11 or Pcol85.

Fig. 4

4C-E ; Pxn expression reveals hemocyte differentiation in the secondary lobes. In E and possibly C, there is an anterior Pxn cluster, close to the PSC. How do the authors interpret this cluster ?

pCol(85), another driver than GMR 13A11 is specifically used for Fig. 4A-F. Why ? Why using two different drivers in separate experiments. At minimum, the relative dynamics of GMR13A11 and pCol expression in the tertiary lobes should be compared, and compared to that of Col/Kn It is essential to add Col antibody stainings to panels A-E

Ubx and sar1/hh (sup fig.4) loss of function reveal a non autonomous (niche) role of Ubx on progenitors in secondary lobes. Ectopic Ubx gain of function in secondary lobes leads to conclude to a cell-autonomous role in preserving progenitors. Could the authors discuss this apparent contradiction !

The number of « Kn/Col expressing cells is also very increased in secondary lobes upon ectopic Ubx expression. Could the authors comment on this.

Fig. 5S D-F. I am not sure of how authors interpret the cell-autonomous effect of Ubx overexpression in Ubx positive cells. Do the Ubx levels significantly drop upon infestation ? Add a secondary lobe specific driver

Fig.6 The title and paragraph, lines 354-381 is very confusing. Does the late-restricted Ubx/Col expression domain correspond to a « niche » or to a « discrete progenitor pool », lines 358. This needs to be clarified My understanding of the cell-tracing experiments is that these cells are niche cells, not or rarely found in adult hematopoietic hubs, while some progenitors expressing Col earlier might contribute to adult hemocytes. They express progenitor markers in early pupae, but what about in adults ? To my best knowledge, there is no evidence for mitotically active hematopoietic cells in adults (Sanchez Bosch et al., 2019). BrdU experiments (Ghosh et al., 2015, 2018) did not rule out endocycling of post-mitotic cells. The new experiments by Kanwal et al., raise the question of the role of the late-traced cells in adults. This point should be considered in the discussion.

Figure 6 itself is confusing

Fig.6 itself. Panel A is particularly confusing. Non only should it be placed after the experimental data panels bu also explained. The adult « fate » of the secondary lobe and posterior tertiary lobe cells should be distinguished from the fate of the Kn/Ubx anterior cells of the tertiary lobes.

A summary figure of the new findings is absolutely necessary.

Minor comments ; suggestions for improvement.

Introduction

The authors cite Rodrigues et al, 2020 who also study secondary and tertiary lobe progenitors in pre-pupae, based on expression patterns. There is some discrepancy between Rodrigues and the submitted article on the expression patterns of HhGal4 and SerGal4. Please clarify this. I would ne nicely complemented by looking at GMRA11>Ubx-RNAi LGs.

Compartimentalisation of the niche (PSC) by ECM proteins was first documented by Krzemien et al., 2010. This and the compartimentalisation of the tertiary and secondary lobes raise the question of the mode of communication between ECM-separated groups of cells,a topic which could possibly be underlined in the discussion.

Results.

All figures

Indicating Pxn, Kn/Col, Dad etc, in red on the figure panels is misleading. What is detected is Pxn-YFP and GMR13A11>mCD8GFP, etc, expression, not the endogenous protein expression. For other reporters, the labelling is correct. Please chek all figures for consistency.

Fig2S. What is the colPS mutation which is not listed among fly stocks? Is it col1 ? In this case please refer to is as col1.

2S E-J’: ColPS posterior lobes F , I, J are much larger than wild type; please comment.

In G, the tertiary lobe is drawn without evidence from DAPI staining ?

Fig. S3 The level of Ser expression in tertiary lobes seems more similar to Ser expression in primary lobes outside the PSC in early third instar (Crozatier et al., 2004 ; Spratford et al., BioRxiv, 2020) than in the PSC. Unlike frc, Ser and Lamin-C are expressed in the entire tertiary lobes, and not restricted to the posterior domain.

Fig. 4E There is a cluster of Pxn positive cells in the posterior tertiary lobe. It would be better to show a detail of this entire lobe in F.

Fig. S5, lines347-349 : *In contrast, increased gstD-GFP levels were observed in the Ubx domain upon wasp infection, in a manner identical to that of Antp expressing PSC/niche (Fig. 5S K-K′, L).* The level of gstD remains low in Ubx cells upon wasp infestation, compared to other cells and it is not possible to compare with Antp cells on this figure.
